# A parasite odyssey: An RNA virus concealed in *Toxoplasma gondii*

**DOI:** 10.1093/ve/veae040

**Published:** 2024-05-11

**Authors:** Purav Gupta, Aiden Hiller, Jawad Chowdhury, Declan Lim, Dillon Yee Lim, Jeroen P J Saeij, Artem Babaian, Felipe Rodriguez, Luke Pereira, Alejandro Morales-Tapia

**Affiliations:** The Woodlands Secondary School, 3225 Erindale Station Rd,Mississauga, ON L5C 1Y5, Canada; Department of Molecular Genetics, University of Toronto, 1 King’s College Circle, Toronto, ON M5S 1A8, Canada; The Donnelly Centre for Cellular + Biomolecular Research, University of Toronto, 160 College St, Toronto, ON M5S 3E1, Canada; The Woodlands Secondary School, 3225 Erindale Station Rd, Mississauga, ON L5C 1Y5, Canada; Department of Molecular Genetics, University of Toronto, 1 King’s College Circle, Toronto, ON M5S 1A8, Canada; The Donnelly Centre for Cellular + Biomolecular Research, University of Toronto, 160 College St, Toronto, ON M5S 3E1, Canada; The Woodlands Secondary School, 3225 Erindale Station Rd, Mississauga, ON L5C 1Y5, Canada; Department of Molecular Genetics, University of Toronto, 1 King’s College Circle, Toronto, ON M5S 1A8, Canada; The Donnelly Centre for Cellular + Biomolecular Research, University of Toronto, 160 College St, Toronto, ON M5S 3E1, Canada; The Woodlands Secondary School, 3225 Erindale Station Rd, Mississauga, ON L5C 1Y5, Canada; Department of Molecular Genetics, University of Toronto, 1 King’s College Circle, Toronto, ON M5S 1A8, Canada; The Donnelly Centre for Cellular + Biomolecular Research, University of Toronto, 160 College St, Toronto, ON M5S 3E1, Canada; The Woodlands Secondary School, 3225 Erindale Station Rd, Mississauga, ON L5C 1Y5, Canada; The Woodlands Secondary School, 3225 Erindale Station Rd, Mississauga, ON L5C 1Y5, Canada; Department of Physiology, Anatomy and Genetics, University of Oxford, Sherrington Building, Sherrington Road, Oxford, Oxfordshire, OX1 3PT, UK; The Woodlands Secondary School, 3225 Erindale Station Rd, Mississauga, ON L5C 1Y5, Canada; Department of Pathology, Microbiology and Immunology, School of Veterinary Medicine, University of California, 1 Shields Ave, Davis, CA 95616, USA; Department of Molecular Genetics, University of Toronto, 1 King’s College Circle, Toronto, ON M5S 1A8, Canada; The Donnelly Centre for Cellular + Biomolecular Research, University of Toronto, 160 College St, Toronto, ON M5S 3E1, Canada; The Woodlands Secondary School, 3225 Erindale Station Rd, Mississauga, ON L5C 1Y5, Canada; The Woodlands Secondary School, 3225 Erindale Station Rd, Mississauga, ON L5C 1Y5, Canada; Department of Pathology, Microbiology and Immunology, School of Veterinary Medicine, University of California, 1 Shields Ave, Davis, CA 95616, USA; Department of Molecular Genetics, University of Toronto, 1 King’s College Circle, Toronto, ON M5S 1A8, Canada; The Donnelly Centre for Cellular + Biomolecular Research, University of Toronto, 160 College St, Toronto, ON M5S 3E1, Canada; The Woodlands Secondary School, 3225 Erindale Station Rd, Mississauga, ON L5C 1Y5, Canada; Department of Molecular Genetics, University of Toronto, 1 King’s College Circle, Toronto, ON M5S 1A8, Canada; The Donnelly Centre for Cellular + Biomolecular Research, University of Toronto, 160 College St, Toronto, ON M5S 3E1, Canada; The Woodlands Secondary School, 3225 Erindale Station Rd, Mississauga, ON L5C 1Y5, Canada

**Keywords:** virus discovery, viromics, RNA viruses, apocryptoviruses, narnaviridae, toxoplasma gondii, apicomplexa, hypervirulence, computational virology

## Abstract

We are entering a ‘Platinum Age of Virus Discovery’, an era marked by exponential growth in the discovery of virus biodiversity, and driven by advances in metagenomics and computational analysis. In the ecosystem of a human (or any animal) there are more species of viruses than simply those directly infecting the animal cells. Viruses can infect all organisms constituting the microbiome, including bacteria, fungi, and unicellular parasites. Thus the complexity of possible interactions between host, microbe, and viruses is unfathomable. To understand this interaction network we must employ computationally assisted virology as a means of analyzing and interpreting the millions of available samples to make inferences about the ways in which viruses may intersect human health. From a computational viral screen of human neuronal datasets, we identified a novel narnavirus *Apocryptovirus odysseus* (Ao) which likely infects the neurotropic parasite *Toxoplasma gondii*. Previously, several parasitic protozoan viruses (PPVs) have been mechanistically established as triggers of host innate responses, and here we present *in silico* evidence that Ao is a plausible pro-inflammatory factor in human and mouse cells infected by *T. gondii. T. gondii* infects billions of people worldwide, yet the prognosis of toxoplasmosis disease is highly variable, and PPVs like Ao could function as a hitherto undescribed hypervirulence factor. In a broader screen of over 7.6 million samples, we explored phylogenetically proximal viruses to Ao and discovered nineteen *Apocryptovirus* species, all found in libraries annotated as vertebrate transcriptome or metatranscriptomes. While samples containing this genus of narnaviruses are derived from sheep, goat, bat, rabbit, chicken, and pigeon samples, the presence of virus is strongly predictive of parasitic *Apicomplexa* nucleic acid co-occurrence, supporting the fact that *Apocryptovirus* is a genus of parasite-infecting viruses. This is a computational proof-of-concept study in which we rapidly analyze millions of datasets from which we distilled a mechanistically, ecologically, and phylogenetically refined hypothesis. We predict that this highly diverged Ao RNA virus is biologically a *T. gondii* infection, and that Ao, and other viruses like it, will modulate this disease which afflicts billions worldwide.

## Introduction

RNA virus discovery is undergoing a revolutionary expansion in the characterization of virus diversity (Shi et al. 2016, 2018; Wolf et al. 2020; Charon et al. 2022; Edgar et al. 2022; Neri et al. 2022; Zayed et al. 2022; Forgia et al. 2023; Hou et al. 2023; Lee et al. 2023; Olendraite, Brown, and Firth 2023; Zheludev et al. 2024). Of the predicted ${10^8}$ to ${10^{12}}$ virus species on Earth (Koonin et al. 2020), $ \approx 300,000$ mammalian viruses are thought to have human-infecting potential (Anthony et al. 2013), of which we know ∼160 RNA viruses (Woolhouse and Adair 2013). This bulk of unknown zoonotic viruses (i.e., viruses that can transmit between non-human animals and humans) are expected to cause the majority of emerging infectious diseases in humans (Jones et al. 2008), with precedent set by the 1918 Spanish influenza, AIDS, SARS, Ebola, and more recently COVID-19. This establishes the real and continued threat that viral zoonoses pose to global health.

Most established relationships between a disease and its causal RNA virus are direct and proximal to infection and are thus tractable to reductionist interrogation. Yet it is evident that confounding variables can impede linking cause and effect: virus genetic heterogeneity, chronic infections, asymptomatic carriers, prolonged latency periods, and microbiome interactions all add complexity to the link between virus and disease. An indirect, yet causal, relationship is well exemplified by Epstein-Barr virus and multiple sclerosis (EBV-MS). The EBV-MS association has long been statistically postulated, yet clear evidence for causality was only established recently, on a background of increasing awareness of the role of neuroinflammation in neurodegeneration (Bjornevik et al. 2022; Lanz et al. 2022; Soldan and Lieberman 2023). Thus, statistical and computational inferences, while not sufficient to formalize causation, do allow for a radical simplification of the space of plausible hypotheses, and thereby accelerate the time to discovery. We opine that in addition to virus discovery, virus effect inference, pathological and ecological, should be a primary objective of the emerging field of computational virology.

### Asking an old question in a new way

The volume of freely available sequencing data in the Sequence Read Archive (SRA) has grown explosively for over a decade (Katz et al. 2021). Currently, there are in excess of 52.96 petabases (Pbp) of freely available sequencing data, capturing $ > 30$ million biological samples. The emerging field of petabase-scale computational biology strives to analyze the totality of these data and enable expansive meta-analyses. The SRA-STAT project has processed over 10.8 million datasets (27.9 Pbp) using a k-mer hashing algorithm to create an index of reads matching known taxa genomes (Katz et al. 2021). Likewise, serratus, which is aimed at uncovering known and novel RNA viruses using a translated nucleotide search for the RNA-dependent RNA polymerase (RdRp), the hallmark gene of RNA viruses, has processed over 7.5 million RNA sequencing datasets (18.97 Pbp) (Edgar et al. 2022). Our group focuses on leveraging this critical mass of freely available data to re-interrogate fundamental questions in virology using a data-driven philosophy. This approach allows us to minimize *a priori* bias and maximize the discovery of unexpected biology. Our immediate focus is to characterize highly divergent neuroinflammatory RNA viruses—these, we hypothesize, have the potential to cause or contribute to human neurological diseases of unknown etiology.

### As simple as it gets—the *Narnaviridae*

One group of poorly understood yet highly divergent viruses are the *Narnaviridae*. This clade of viruses are among the simplest viruses, comprising a naked, +ve sense RNA genome (hence the derivation from ‘naked RNA’) encoding an RNA-dependent RNA polymerase; the virus is thus observed as a ribonucleoprotein complex, with no true virion (Hillman and Esteban 2011; Hillman and Cai 2013). Members of this family of viruses are best known for their association with fungi, and indeed the first two species identified, Saccharomyces 20S RNA virus (ScNV-20S) and Saccharomyces 23S RNA virus (ScNV-23S), were discovered in the model organism *Saccharomyces cerevisiae* (Kadowaki and Halvorson 1971; Garvik and Haber 1978; Wejksnora and Haber 1978). ScNV-20S and ScNV-23S infections are mostly not associated with a phenotype (Hillman and Esteban 2011; Hillman and Cai 2013), although, as is the case with the *S. cerevisiae* L-A virus, chronic apathogenic infections may become phenotypic in specific genetic backgrounds (Chau et al. 2023). Likewise, although *S. cerevisiae* strains harboring high expression of a related narnavirus, N1199, display defects in sporulation, autophagy, and a change in metabolite utilization, strains with a low N1199 expression are more common and display no phenotype (Vijayraghavan et al. 2023). A comparison of virus-infected and virus-eliminated strains of *Aspergillus flavus* found that narnavirus infection is not associated with changes in colony appearance, growth rate, or mycelial/hyphal morphology, despite changes in transcriptomic profile (Kuroki et al. 2023). Besides fungi, *Narnaviridae* have also been found in marine protists (Charon, Murray, and Holmes 2021; Chiba et al. 2023), mosquitoes (Batson et al. 2021; Abbo et al. 2023; Yang et al. 2023), and other arthropods (Harvey et al. 2019; Xu et al. 2022).

### Parasitic protozoan viruses, nested invaders

The niches of the human virome extend beyond human cells; our holobiont constitutes an array of biological hosts including the bacteria, fungi, plants, and parasites. Of interest is the capacity of diverse viruses to modulate the physiology of these non-human hosts and in doing so, influence the biology of the ‘macrohost’—*Homo sapiens* (Gómez-Arreaza et al. 2017; Heeren et al. 2023; Zhao et al. 2023). Perhaps unsurprisingly, bacteria and their bacteriophages dominate the human microbiome and have been the focus of the majority of metagenomic research to date (Khan Mirzaei et al. 2021; Liang and Bushman 2021). Yet one intriguing category of human-adjacent viruses are parasitic protozoan viruses (PPVs) which infect the eukaryotic phyla *Euglenozoa* and *Apicomplexa* (Lye et al. 2016; Grybchuk et al. 2018; Charon et al. 2019; Rodrigues, Roy, and Sehgal 2022). PPVs are a diverse functional, rather than phylogenetic, grouping and are generally poorly characterized (Gómez-Arreaza et al. 2017; Heeren et al. 2023; Zhao et al. 2023). More than mere passengers, the presence of a PPV within a parasite and subsequent exposure of the macrohost to PPV-derived pathogen-associated molecular patterns (PAMPs) can modulate macrohost immune responses, with important implications for pathogenicity (Gómez-Arreaza et al. 2017; Zhao et al. 2023). Viral PAMPs, including viral RNA (vRNA), are sufficient to initiate an innate immune response via nucleic acid sensors (NAS), even in the context of parasitic infection of the macrohost. NAS include toll-like receptors (TLRs) which survey endosomes for dsRNA (TLR3), ssRNA (TLR7,8), or unmethylated CpG motifs in ssDNA (TLR9) (Fitzgerald and Kagan 2020). Additionally, the three RIG-I-like receptors (RLRs)—the signaling RIG-I and MDA5, and the regulatory LGP2—survey the cytosol for vRNA transcripts with an exposed 5ʹ triphosphate or misprocessed cellular RNA (Rehwinkel and Gack 2020). Collectively, NASs orchestrate an antiviral type I interferon (IFN) response (Lee and Ashkar 2018). For example, the dsRNA virus Cryptosporidium parvum virus 1 (CSpV1), which infects *Cryptosporidium parvum*, activates a type I IFN inflammatory cascade in mouse and cell culture models. Interestingly, this response undermines host defences against *C. parvum*, as evidenced by the enhanced antiparasitic immunity observed in mice lacking type I IFN receptor in their intestinal epithelia (Deng et al. 2023). While the underlying mechanism for the inflammation remains elusive, PPV presence appears to be necessary for parasite pathogenicity, potentially by diverting the host’s innate immune system toward the activation of antiviral immunity and away from antiparasitic immunity. The activation of a NAS-dependent type I IFN response by a PPV is also observed with Trichomonas vaginalis virus (TVV) infecting *Trichomonas vaginalis* and Leishmania RNA virus infecting *Leishmania sp*. (Fichorova et al. 2012; de Carvalho et al. 2019; Narayanasamy et al. 2022). In both of these common human pathogens, the virus is predicted or observed to worsen the severity of parasitic disease. Conversely, PPVs, such as Giardia lamblia virus 1 (GLV1), can also impair parasitic pathogenesis. In the case of GLV1, the virus inhibits the growth of *G. lamblia* (Miller, Wang, and Wang 1988), highlighting the complexity of this tripartite relationship. No such relationships, however, have been observed in narnavirus or narnavirus-like viruses.

Surprisingly, there are no known viruses associated with *Toxoplasma gondii* (*T. gondii*), an apicomplexan parasite that infects approximately 30 per cent of the global human population, with some geographic regions reaching majority seroprevalence (Pappas, Roussos, and Falagas 2009; Dubey 2021; Bisetegn et al. 2023). *T. gondii* has a notably broad host range of warm-blooded animals and tropism for all animal tissues, including the brain (Pappas, Roussos, and Falagas 2009). While *T. gondii* infection in *Homo sapiens* is most commonly asymptomatic and self-limiting, it remains a dangerous, opportunistic infection in immunocompromised patients and pregnant women, and a leading infectious cause of blindness (Khurana and Batra 2016; Goh et al. 2023). Furthermore, sporadic *T. gondii* strains are hypervirulent, with the ability to cause severe disease even in immunocompetent individuals (Dardé et al. 2020). Thus, the full global health burden of toxoplasmosis remains unclear, especially its possible role in chronic neuroinflammation and subsequent neurodegenerative disease.

### Tell me, O Muse—a viral odyssey

In this work, we use the SRA and serratus to screen for potential human neuroinflammatory viruses, identifying *Apocryptovirus odysseus*, a narnavirus hidden within *Toxoplasma gondii*, and nineteen additional members of the proposed genus *Apocryptovirus*, all of which likely infect apicomplexan parasites. We establish these viruses in their phylogenetic context, estimate their prevalence, and perform sequence and structure analysis of the *Apocryptovirus* RdRp. Finally, we provide computational evidence and describe a model for *Apocryptovirus odysseus*-mediated *Toxoplasma* hypervirulence.

## Results

### Identification of T. gondii-associated viral sequences

**Figure 1.**
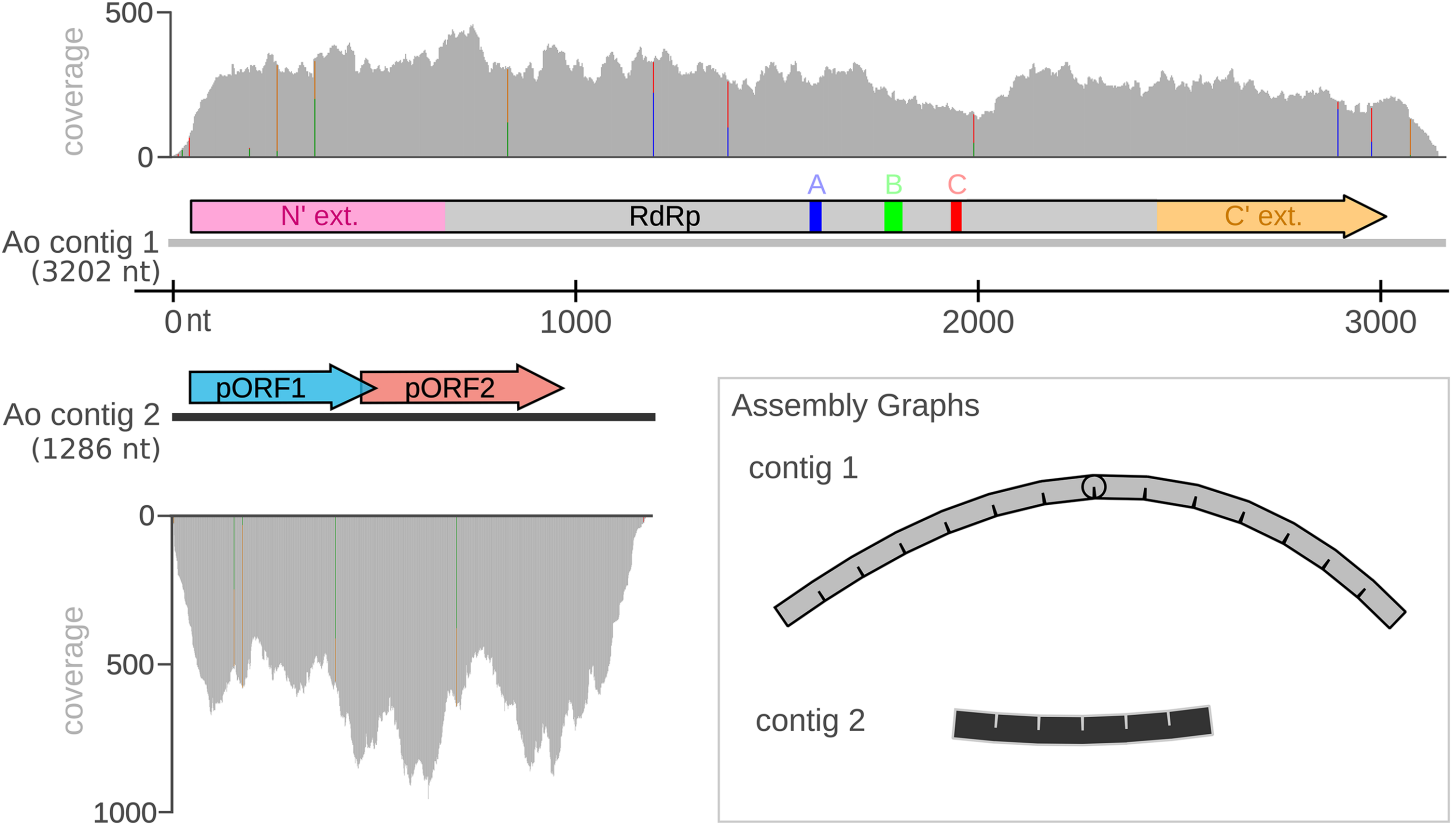
*Apocryptovirus odysseus* (Ao) genome. From (rnaspades 3.15.5) assembly of the Ngô RNA sequencing dataset (SRR1205923), we recovered a high-coverage (123× coverage) RdRp-encoding contig, and identified a second correlated contig of likely viral origin. Contig 2 (279× coverage) contains two putative ORFs: pORF1 and pORF2, but these ORFs do not have identifiable homologs via BLASTp in the non-redundant and transcriptome shotgun assembly databases (date accessed: June 2023), TBLASTN with the nucleotide database, or by InterProScan or HHpred (date accessed: June 2023). Inlay, unbranched assembly graphs (Bandage 0.8.1) of both contigs confirmed a linear genome structure.

Using serratus to screen for novel human neuroinflammatory RNA viruses, we searched the BioSample database (Barrett et al. 2012) for SRA libraries annotated with metadata describing the sample as (1) human and (2) central nervous system (see ‘Materials and methods’ section). From the 7,675,502 sequencing runs or 18.97 Pbp processed in the serratus database, 483,173 (6.29 percent) runs were annotated as central nervous system samples, of which 82,454 (17.07 per cent) were annotated as human. We further filtered to ‘neuron’ annotated datasets containing an unknown virus ($ \le $90 per cent RdRp amino acid identity as detected by palmscan (Babaian and Edgar 2022), which yielded a shortlist of ten matching libraries. By contig coverage, three of the highest-expression libraries (SRR1205923, SRR1204654, and SRR1205190) originated from one BioProject (PRJNA241125), which we manually inspected to identify a divergent RdRp fragment (palmprint: u150420_halalDiploma). We have termed the BioProject’s associated paper ‘the Ngô study’, wherein the authors infected three human cell types (neuronal stem cells, neuronal differentiated cells, and monocytes) *in vitro*, with nine different strains of the apicomplexan parasite *T. gondii* or a mock control (Ngô et al. 2017). We assembled all 117 available Ngô mRNAseq runs *de novo* and with palmscan identified a 3,177 nucleotide (nt) putative viral contig with a coding-complete 989 amino acid RdRp (Fig. 1). A blastp search on the translated protein revealed highly divergent RdRp homologs; the highest identity match was an unclassified *Riboviria* RdRp (date: 18 June 2023, accession: QIM73983.1, percent-identity: 54.13 per cent, query-coverage: 40 per cent, e-value: 9e-133).

**Figure 2.**
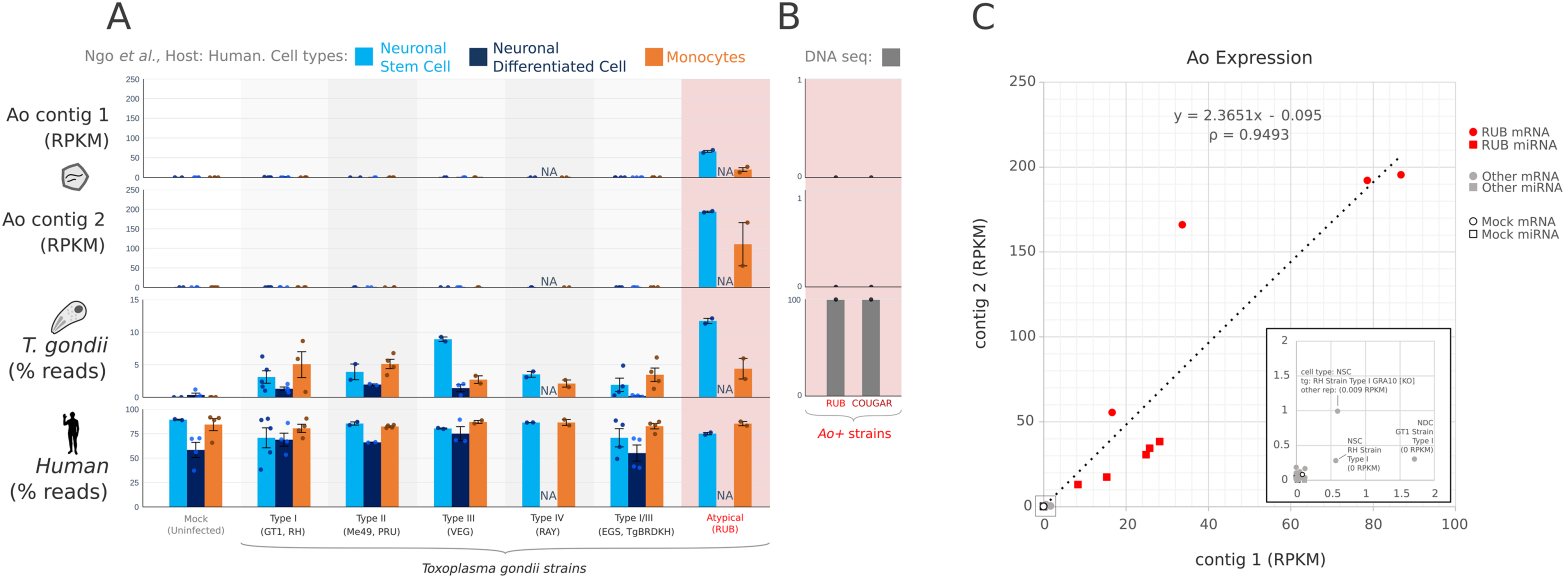
Quantification of *A. odysseus* (Ao) genome across Toxoplasma-infected human cell lines in the Ngô study. (A) The expression (reads per million kilobase of mapped reads, RPKM) of contig 1 and contig 2 of *A. odysseus* (Ao) was quantified in three cell types (*n* = 37 neuronal stem cell, NSC; *n* = 24 neuronal differentiated cell, NDC; and *n* = 40 monocytes), infected by one of nine strains (*n* = 117 runs total) of *T. gondii*, in addition to quantifying Toxoplasma or human genome-mapped reads (percentage total) from the same datasets. Ao was found at high expression exclusively in *T. gondii*—RUB strain samples within Ngô study mRNAseq and miRNAseq (not shown). Data points and 2 SD Error Bars are shown. (B) DNAseq quantification of Ao in four replicates of *T. gondii*—RUB (0/40,151,652 reads) and two *T. gondii*—COUGAR (0/31,562,801 reads) in BioProject: PRJNA61119 fails to identify any virus mapping reads. (C) Ao contig 1 and contig 2 expression (reads per kilobase of million mapped reads, RPKM) and Pearson correlation between contig 1 and contig 2 expression across all mRNA and miRNAseq datasets in the Ngô study, *N* = 237.

We then screened for additional viral segments or co-occurring contigs by depleting contigs mapping to the genomes of either *T. gondii* or *H. sapiens* (‘Identification of Ao contig 2 via host read-depletion’). This produced a second 1,283 nt contig whose expression correlated with the RdRp (Pearson correlation $R = 0.95$) (Fig. 2C). This contig encodes two open reading frames (ORFs) with no identifiable homologs (Fig. 1). Given that the well-conserved RdRp sequence is already highly divergent, it is not unexpected that a viral accessory or structural gene would prove more difficult to identify based on sequence homology.

To quantify the relative abundance of human, parasite, and virus nucleic acids across each library, we aligned the reads against each respective genome and observed that high expression of the putative viral RdRp contig (RPKM $ \ge 10$) was specific to samples experimentally infected by the *T. gondii*—RUB strain (mRNAseq = 4/4, miRNAseq 5/5), and was mostly absent (RPKM $ \le 1.0$) from other strains or controls (mRNAseq = $112/113$, miRNAseq $115/115$, Fisher’s Exact Test, *P*  $ < 1e - 4$) (Fig. 2A). We rule out that the RdRp is an endogenous viral element since corresponding DNAseq data have zero virus-aligning reads (Fig. 2B). Trace RdRp expression (RPKM 0.5–2.0) was observed in three *T. gondii*-infected samples, all annotated as other strains: RH, GT1, and RH-GRA10[KO]. The relatively low abundance of sequencing reads is suggestive of sequencing cross-contamination (sequence batch information, Supplementary Table S1), although early stages of viral cross-infection between samples cannot be ruled out, in which case the RUB strain would be a likely source (Fig. 2C).

We propose that these two contigs constitute the genome of a bi-segmented RNA virus infecting *T. gondii*—RUB, which we name *Apocryptovirus odysseus* (Ao). The genus name derives from a Greek root suggesting a hidden or concealed virus, while we derive the species name from the leader of the soldiers who hid within the Trojan Horse in Greek myth, who when revealed wreaked havoc in the city of Troy.

### *A. odysseus* infects geographically distinct strains of *T. gondii*

Next, we investigated the global distribution of *T. gondii* and more broadly *Apicomplexa*-containing sequencing data and their association, if any, with apocryptoviruses.

**Figure 3.**
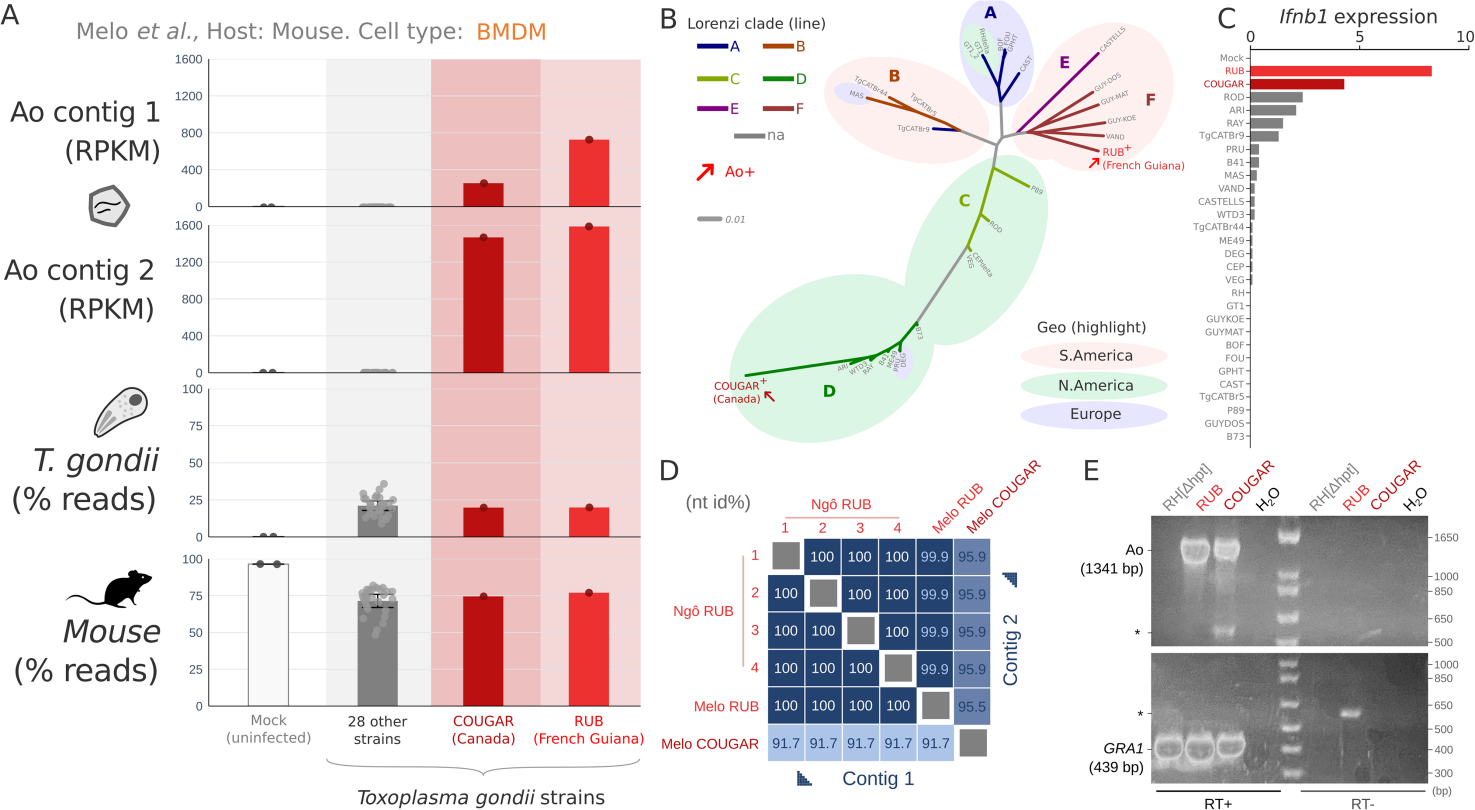
Quantification of *A. odysseus* in Melo et al. (A) Quantification of Ao-aligning reads amongst thirty strains of *T. gondii* strains and mock control, error bars indicate 2 SD. (B) Unrooted *T. gondii* strain phylogeny (IQ-TREE) from transcriptome in the Melo study (149,777 measured sites; nodes have 100 per cent bootstrap support, extended in Supplementary Fig. S1), and strain cross-contamination ruled out by transcriptome variant analysis in Supplementary Fig. S2. The original geographic isolation of strains is highlighted as North American, European, or South American, and lines are colored by the genomic *T. gondii* clades defined by Lorenzi et al. (Supplementary Table S2). *A. odysseus* (Ao, red arrow) was detected at high levels in two geographically and phylogenetically unrelated strains of *T. gondii*; RUB isolated from a symptomatic soldier in French Guiana in 1991 and COUGAR isolated from a cougar in British Columbia in 1996. (C) RNA sequencing expression data obtained from Supplementary Table S1 of the Melo et al. study showing normalized expression levels of Ifnb1 in murine macrophages infected with strains of *T. gondii*. (D) Pairwise nucleotide sequence identity of complete coding sequences from contig 1 and 2 from the Ngô and Melo studies. (E) Reverse Transcriptase (RT) PCR for Ao or GRA1, a highly expressed Toxoplasma gene used as an RNA quality control, from Ao+ (RUB and COUGAR) and Ao—control RH[delta hpt] strains of Toxoplasma grown on HFFs. Asterix denotes non-specific bands. Sanger sequencing of amplicons showed 100 per cent nucleotide identity to the assembled Ao RUB and Ao COUGAR, respectively.

We hypothesized that Ao would only be observed if its parasite host was present. To test this, we re-queried all 7.5 million sequencing runs processed by serratus for high-similarity matches to Ao ($ \ge $90 per cent aa-identity) and identified one additional Ao+ BioProject (0.00052 per cent, or 1/191,678 BioProjects), PRJNA114693. In the associated publication (‘the Melo study’), the authors infected murine macrophages *in vitro* with thirty distinct strains of *T. gondii*, or a mock control (Melo et al. 2013). Following *de novo* assembly of all thirty-two Melo samples, we identified both Ao contigs in two samples: again a *T. gondii*—RUB strain (SRR446933), and *T. gondii*—COUGAR strain (SRR446909). Ao contig 1 and contig 2 exhibited a high expression (RPKM $ \ge 10$) in RUB and COUGAR libraries, and were absent (RPKM $ \le 1$) from twenty-eight other *T. gondii* strains or the mock control (Fig. 3A). To assess whether Ao positivity in RUB and COUGAR could be explained by genetic similarities between the two strains, we performed a phylogenetic analysis of the *Toxoplasma* parasite. Over the estimated 1 million years since *T. gondii* arose (Bertranpetit et al. 2017), the parasite has radiated into phylogeographically distinct clades A through F (Lorenzi et al. 2016). *T. gondii*—RUB and COUGAR strains are phylogenetically and geographically divergent from one another; RUB is a clade F strain isolated from a human in French Guiana (Dardé et al. 1998), while COUGAR is a clade D strain isolated from a mountain lion associated with a toxoplasmosis outbreak in British Columbia, Canada (Aramini, Stephen, and Dubey 1998) (Fig. 3B), yet as noted by Melo *et al*., these unrelated strains are both strong inducers of interferon beta (*Ifnb1*) (Fig. 3C). To identify additional RUB or COUGAR RNA sequencing data, we performed a metadata search but failed to find any additional datasets in the SRA from these strains. Given the available data, Ao appears to be fully penetrant amongst *T. gondii* RUB and COUGAR RNAseq libraries. Comparing the Ao RdRp coding sequences across samples, the Ao RUB from Ngô and Ao RUB from Melo had 100 per cent nucleotide identity (nt id), while the Ao RUB and Ao COUGAR had only 91.7 per cent nt id. There is no read-level support for cross-contamination between *T. gondii* RUB and COUGAR samples (Fig. S2), which, combined with the cross-sample Ao virus sequence variation, supports the fact that the Ao RUB and Ao COUGAR are distinct strains of the virus. Further, Ao viral RNA was confirmed by reverse transcriptase polymerase chain reaction in *T. gondii* cultured on human fibroblasts in the RUB and COUGAR strains, and were absent in RH[delta hpt] strain, and without the addition of RT (control) (Fig. 3E). Finally, we also performed a BLASTn search of the Ao COUGAR RdRp contig using the BLAST expressed sequence tag (est) database (accessed: 22 February 2024), and identified fifteen ESTs (top hit: CV549349.1, mean nt id: 98.3 per cent, max e-value: 2e-90, size range: 189–805 nt, sample submission date 1 July 2004, query: Ao RUB, mean nucleotide identity: 90.4 per cent) which were all isolated from *T. gondii* COUGAR tachyzoites (Ajioka et al. 1998). Likewise, searching for Ao COUGAR contig 2 in the est database revealed seventy-two ESTs (top hit: CV653441.1, mean nt id: 99.1 per cent, max e-value: 8e-56, size range: 165–601 nt, query Ao RUB, mean nt-id: 93.85 per cent). The available samples of RUB and COUGAR are experimental/laboratory-associated; therefore, it is undetermined if Ao is found in wild isolates. Regardless, given the evolutionary, geographic source, and *T. gondii* host strain differences between the Ao RUB and COUGAR viral strains, and the absence of virus in evolutionary intermediate *T. gondii* strains, it is plausible that Ao is a horizontally infectious virus circulating in *T. gondii*.

### Apocryptoviruses are a diverse clade of parasite-associated narnaviruses

To elucidate the evolutionary and ecological context of Ao, we interrogated the viruses closely related to the virus. Although hidden Markov model (HMM) homology search via pfam and interproscan (Finn et al. 2014; Jones et al. 2014) failed to recognize the Ao RdRp (date accessed: June 2023), remote homologs were identified with blastp within the *Narnaviridae* (date accessed: June 2023). This discrepancy is likely due to a lack of adequate narnavirus representation in standard HMMs: Ao shares only 25 per cent and 28 per cent amino acid identity with the exemplar narnaviruses ScNV-20S and ScNV-23S, respectively. The highest similarity match to Ao RdRp was an unclassified ribovirus partial RdRp (52 per cent aa identity, query coverage 40 per cent, subject coverage 100 per cent accession: QIM73983.1) isolated in 2016 from the lung of a Ryukyu mouse (*Mus caroli*) in Thailand (Wu et al. 2021). The remaining narnavirus matches were distal, at below 40 per cent amino acid identity.

**Figure 4.**
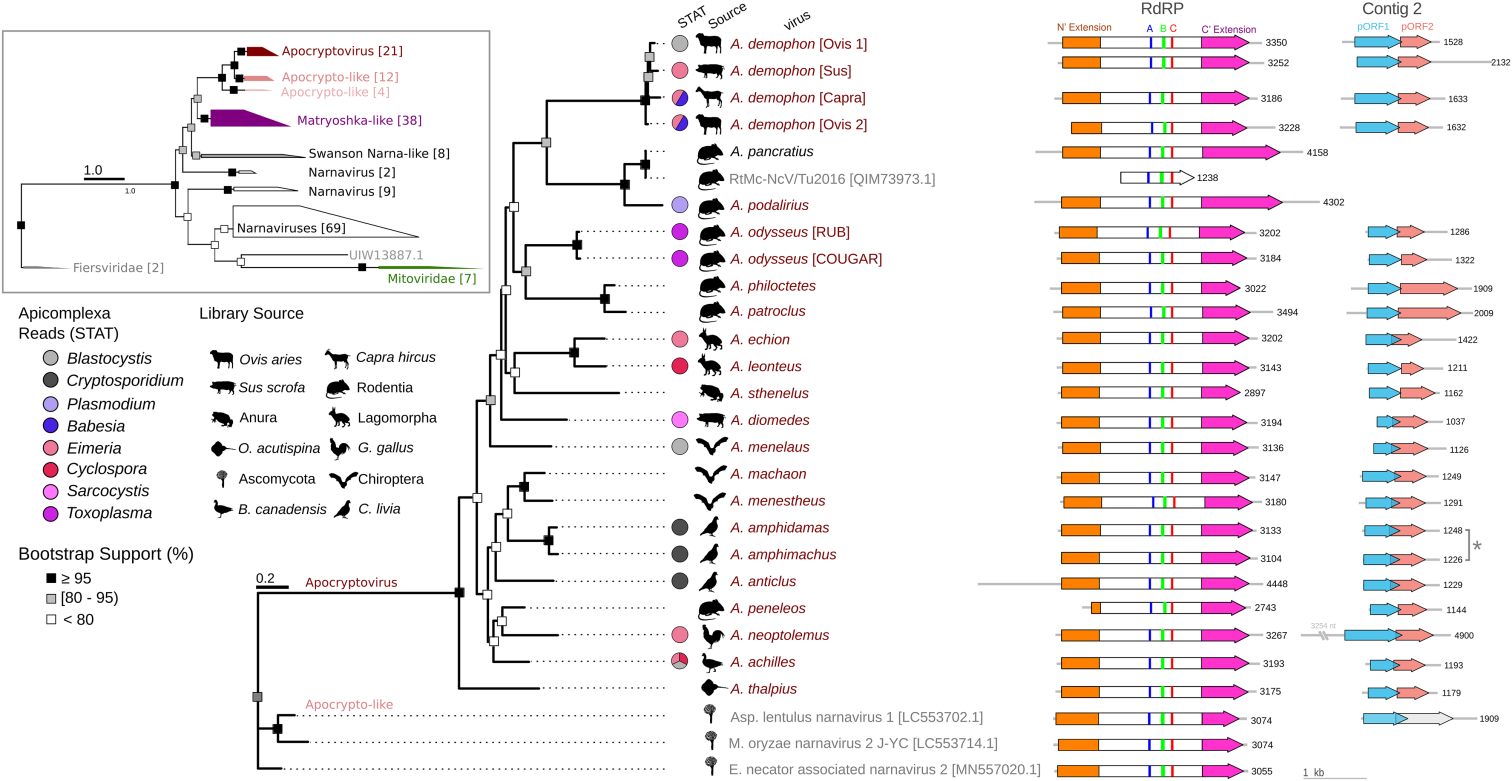
*Apocryptovirus* RdRp phylogeny and genome maps. Maximum-likelihood phylogenetic tree (IQ-TREE, 2.1.4 & ggtree, 3.10.0) and contigs 1 and 2 for the twenty proposed species of Apocryptoviruses estimated based on RdRp palm (motifs F-E) and thumb subdomains, with an outgroup of representative Apocrypto-like viruses. Inlay shows the placement of Apocryptoviruses within *Narnaviridae* (see: Fig. S4). Novel viruses are named and GenBank viruses have accessions. Scale bars represent amino acid substitutions per site, and square symbols denote bootstrap confidence. For each Apocryptovirus, the source sequencing library taxonomic label (silhouettes), and co-occurring *Apicomplexa*-categorized reads. Conserved RdRp ORF (contig 1) and pORF1,2 contigs with contig length indicated in nucleotides. *A. amphidamas* and *A. amphimachus* were recovered from a common sequencing library; therefore, the corresponding contig 2 is not known with certainty (denoted with asterisk).

Due to a paucity of data within the family *Narnaviridae*, and the divergent nature of Ao, we re-queried serratus for Ao-like RdRp palmprints. We retrieved and assembled 166 matching runs from 46 BioProjects with 32 species-like operational taxonomic units (sOTUs) (186 distinct virus-run observations, Supplementary Table S3) (Chen et al. 2022; Zhou et al. 2021; Gil and Hird 2022; Gao et al. 2021; He et al. 2021; Chen, Wei-Chen, and Shi 2021; Rhie et al. 2021; Bittner, Mack, and Nachman 2022; Chen et al. 2021; Xie et al. 2021; Cui et al. 2022; Wen et al. 2022; Zhou et al. 2022b; Sun et al. 2022; Wu et al. 2021). We recovered relevant narnavirus RdRp contigs for all 32/32 (100 per cent) sOTUs, of which 22/32 (75 per cent) contained a sufficiently long RdRp sequence ($ \approx 390$ amino acids, motif F-E (Venkataraman, Prasad, and Selvarajan 2018) and thumb for robust phylogenetic reconstruction. In addition, we enforced that RdRps must be $ < 90$% identity from other members to be designated as distinct species; otherwise, sequences were considered as strain-level variants. Thus, two pigeon-associated sOTUs (*Columba livia*) were considered as one virus species *A. anticlus; A. pancratius* described here is a strain of the *Ribovirus sp*. (RtMc-NcV/Tu2016) (QIM73973.1) and four domestic animal-associated sOTUs (two *Ovis aries, Capra hircus*, and *Sus scrofa*) were considered as one species: *A. demophon*. In total, this yields twenty species members of the genus *Apocryptovirus* (Fig. 4 and Extended Table S3).

Using both the novel RdRps recovered from the SRA and alignable RdRp hits in GenBank, we constructed a maximum-likelihood phylogenetic tree (Fig. 4). Ao and the Ao-like serratus hits, as well as the rodent lung virome narnavirus, form a monophyletic clade (bootstrap support 100 per cent). These RdRp share at least 70 per cent amino acid identity with another member in the clade, and less than 70 per cent identity with all members outside the clade (Supplementary Fig. S3). We propose that these viruses, together with Ao, constitute a novel genus within *Narnaviridae*. Apocryptoviruses are situated within a larger Apocrypto-proximal clade that also comprises the Matryoshka RNA viruses (Charon et al. 2019), all of which are in *Narnaviridae* with high confidence (Fig. 4). Individual viral species of this new genus have been named after the other soldiers hidden inside the Trojan Horse that were led by Odysseus in the myth.

**Figure 5.**
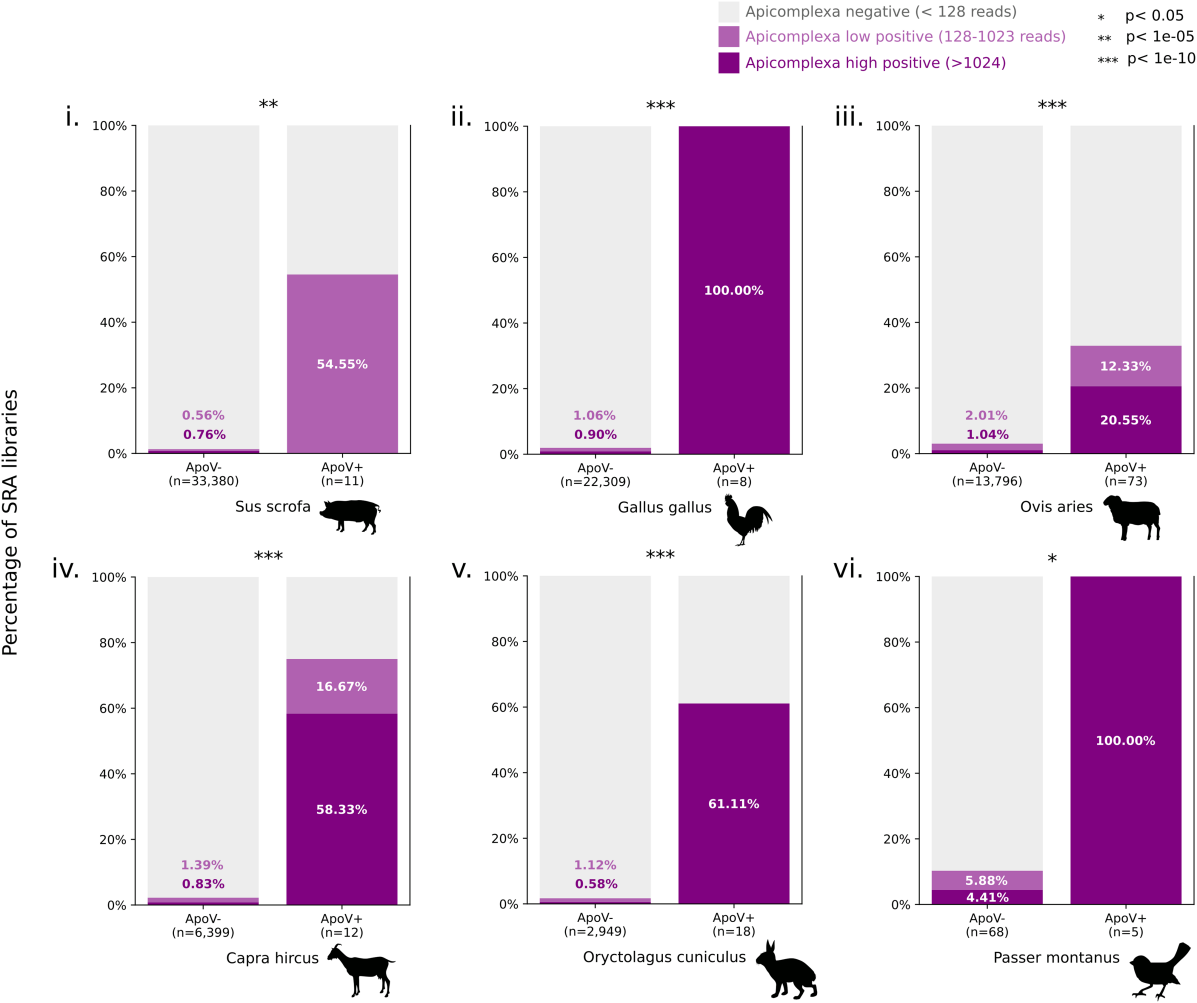
Apocryptoviruses are predictive of *Apicomplexa* co-occurrence. Samples from six non-laboratory animals (i. pig, ii. chicken, iii. sheep, iv. goat, v. rabbit, and vi. tree sparrow) were first categorized as *Apocryptovirus*-negative (ApoV-) or *Apocryptovirus*-positive (ApoV+). We then measured the amount of STAT classified *Apicomplexa*-reads in each library and classified each library as: *Apicomplexa*-negative, ≤128 reads (gray); low *Apicomplexa*-positive, 128–1,023 reads (pale); or high *Apicomplexa*-positive, ≥1024 (dark). The significance of the co-occurrence was evaluated with a Fisher’s Exact Test (Methods), for ApoV± and *Apicomplexa* (high or low)±.

The prevalence of Ao and the Apocryptoviruses is rare amongst SRA samples. Only 6/23,530 (0.025 per cent) runs annotated as *T. gondii* are Ao-positive, and zero runs containing $ \ge 1024$  *Toxoplasma sp*. reads measured by stat (Katz et al. 2021), and not annotated as *T. gondii*, are Ao-positive (0/9,071). The rate of Ao-positivity is estimated to be below 1 per cent in *Toxoplasma*.

Predicting virus host range based on metagenomic data is an ongoing challenge in viromics. The overwhelming majority of *Narnaviridae* are metagenomic or from complex samples such as leaf lesions. The closest identified narnavirus with a plausible host assignment is Aspergillus lentulus Narnavirus 1 (AleNV1, bcH36643.1), isolated from cultured *Aspergillus lentulus* mycelia (Chiba et al. 2021). The next closest are the Matryoshka RNA viruses isolated from human blood co-infected with *Plasmodium* or *Leucocytozoon* parasites (Charon et al. 2019).

Nominally, Ao was identified in libraries labeled as *H. sapiens* (Ngô et al. 2017) or *M. musculus* (Melo et al. 2013), yet text metadata and taxonomic classification of nucleic acids in those libraries pointed us toward *T. gondii* as the common virus-associated factor, suggesting the parasite was the host for Ao. This virus–host relationship was strengthened when Ao was found in multiple replicates specific to 1/9 *Toxoplasma* strains in the Ngô study, and 2/31 strains in the Melo study, as well as in *Toxoplasma* ESTs. Generalizing this rationale, we decided to measure the extent of *Apicomplexa* positivity in each of the Apocryptovirus-positive libraries and found that 10/18 (55 per cent) of the viruses were associated with *Apicomplexa* in at least one dataset (Fig. 4 and Supplementary Table S4).

To further test the relationship between *Apocryptovirus* and *Apicomplexa*, we calculated their rate of co-occurrence per library for each source organism and compared this to the background rate of *Apicomplexa* positivity. We found that apocryptoviruses are highly predictive (Fisher’s exact test) for the co-occurrence of *Apicomplexa* in pig (*P* = 2.42E-09), chicken (*P* = 2.34E-14), sheep (*P* = 2.02E-18), goat (*P* = 3.64E-13), rabbit (*P* = 3.09E-15), and sparrow (*P* = 5.27E-05) (S5). Conversely, we measured the prevalence of apocryptoviruses amongst *Apicomplexa*-positive samples in the SRA and found the viruses occurred variably in pig (6/447, 1.34 per cent virus-positive), chicken (8/445, 1.79 per cent), sheep (24/444, 5.40 per cent), goat (9/151, 5.96 per cent), rabbit (11/61, 18.03 per cent), and sparrow (5/12, 41.66 per cent) samples.

Parsimony suggests apocryptoviruses are infecting *Apicomplexa*, which in turn would allow the viruses to enter, but not infect, vertebrate host cells. Currently there is no evidence that apocryptoviruses can replicate in vertebrates in the absence of an apicomplexan. As such, in any molecular interaction between a virus-infected apicomplexan and the macrohost, the virus is likely a bystander. An alternative hypothesis is that apicomplexan infection sensitizes the vertebrate cells to apocryptovirus infection/replication, but the resolution of these hypotheses will require further experimental validation.

The viruses *A. neoptolemus, A. diomedes*, and (particularly) *A. demophon* showed a high prevalence in parasites associated with livestock, and may thus modulate the biology of agriculturally relevant pathogens. Of note among these is *Eimeria sp*., a causative agent of coccidiosis, a high mortality disease causing billions of dollars of economic loss for farmers (Blake and Tomley 2014).

### Structural characterization of *Apocryptovirus* proteins

**Figure 6.**
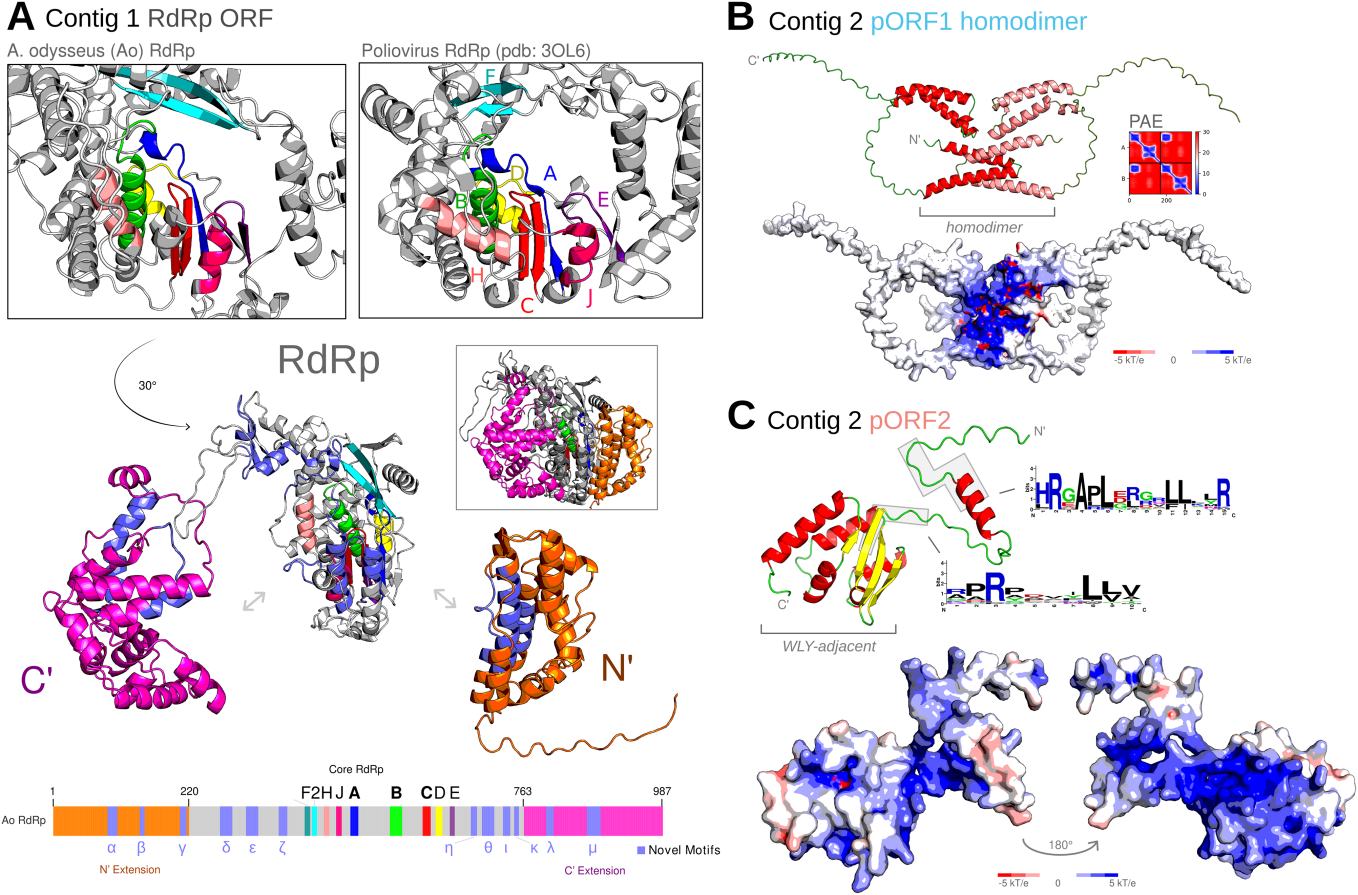
Protein structural analysis. (A) Predicted structure of Ao RUB RdRp and solved X-ray structure of poliovirus RdRp (PDB accession 3OL6 (Gong and Peersen 2010)), at their respective catalytic cores, with motifs F-E highlighted (Venkataraman, Prasad, and Selvarajan 2018). Ao RdRp is shown in an exploded view, rotated with motif C facing forward. The N’ and C’ terminal extensions form distinct domains from the catalytic core RdRp (Supplementary Fig. S5). The Apocryptovirus sequence motifs had similar conservation to the RdRp core motifs against a background of sequences falling outside of the motifs—high occupancy inter-motif sequences (Supplementary Fig. S6). (B) Predicted structure of Ao RUB contig 2 pORF1 as a homodimer, and calculated surface electrostatic potential map (apbs v3.4.1 (Jurrus et al. 2018) in PyMOL v2.5.0 Open Source (Schrödinger 2015)). The predicted aligned error (PAE) shows a high confidence interaction between the N’ alpha helices. (C) Predicted structure and electrostatic potential map of Ao RUB contig2 pORF2, with conserved region sequence logo highlighted.

The structure of narna-like RdRp has not been well characterized. Comparing the predicted structure for Ao RUB RdRp (confident, predicted local distance difference test: 84.56) to the experimentally solved Poliovirus RdRp structure, Ao RdRp folds into a palm superfamily structure with fingers, palm, and thumb (Fig. 6) with intact RdRp sequence motifs (Venkataraman, Prasad, and Selvarajan 2018). We confirmed that the *Apocryptovirus* contig 1 encodes a biochemically competent RdRp on the basis of core RdRp motif conservation (Fig. 6A). We noted that the RdRp of Apocryptoviruses have N’- and C’-extensions of 220 and 224 amino acids long, respectively, that extend beyond the palm, fingers, and thumb of a minimally viable RdRp (with poliovirus being a well-studied exemplar of a minimal RdRp). To investigate the possible activity of these extensions, we first sought to analyze sequence motifs conserved in the Apocrypto-proximal clade, and then apply these to structure. We re-aligned more closely related (Apocrypto-proximal) RdRps, which allowed us to create a higher confidence multiple sequence alignment (MSA) of the full protein sequence, inclusive of the extensions. We identified twelve high-occupancy and highly conserved regions from the MSA and designated them with lowercase Greek letters $\alpha $ through $\mu $ (Supplementary Fig. S5). We reconstructed the MSA for the Ao RUB pORF1 and pORF2 (hmmer, 3.3.2) to improve protein structure prediction with colabfold. The pORF1 has confident predictions for two positively charged helix-turn-helix pairs with intervening disordered loops. We then assessed if this protein could form homodimers; indeed the positive surface charges localize to one another, and the pair of helices interlock in a putative homodimerization domain (Fig. 6). The pORF2 was predicted to be more structured, with an N’ alpha helix coinciding with a conserved positive-charge motif, and a short unknown domain. The linker and alpha-helices of pORF2 have a positively charged surface with well-conserved Arg residues (Fig. 6). Searching for structures similar to pORF2 with foldseek (accessed: 24 February 2024) (Kempen et al. 2023), we uncovered fifteen hits (probability $ \ge $0.95, e-value $ \le $1e-1), thirteen (86.7 per cent) of which are annotated as being WYL domain-containing. This Ao pORF2 domain is itself not a WYL-domain, but pORF2-like domains are found adjacent to WYL. WYL domains can bind nucleic acids and are well characterized to play a role in transcriptional regulation in bacteria (Keller and Weber-Ban 2023); considered together with the conserved positive charges on pORF2, we hypothesize this protein is involved in a nucleic acid interaction. Related narnaviruses have reported second segments: Aspergillus lentulus Narnavirus 1 (Chiba et al. 2021), Matryoshka viruses (Charon et al. 2019), and Leptomonas seymouri Narna-like virus 1 (a PPV) (Sukla et al. 2017), although blastp did not retrieve this as a match. To test for remote homology, we constructed HMMs for *Apocryptovirus* pORF1 and pORF2. The pORF1 (but not the faster evolving pORF2) (Supplementary Fig. S3B), models matched the Aspergillus lentulus Narnavirus 1 ORF1 (hmmscan v3.4, E-value: 4.6e-10, bitscore 26.6) establishing a remote homology between the contig 2 of these related narnaviruses.

### Is *A. odysseus* a *T. gondii* hypervirulence factor?

*T. gondii* RUB and COUGAR are atypically hypervirulent. RUB was isolated in 1991 from an immunocompetent soldier in French Guiana presenting with fever, myalgia, and leukopenia which developed into rales, respiratory failure, and renal deterioration (Dardé et al. 1998). While the COUGAR strain was isolated from a mountain lion (Dubey et al. 2008, 2021), it is believed to be identical to the strain which infected 3,000–7,000 people in a water-borne 1994/1995 toxoplasmosis outbreak in Victoria, BC (Bowie et al. 1997). Notably, the incidence of ocular inflammation (retinitis) amongst these immunocompetent patients was high in the Victoria outbreak (Bowie et al. 1997). Melo *et al.* note that exactly these two ‘atypical’ *T. gondii* strains are outliers by their capacity to induce a type I interferon inflammatory response in murine cells (see also Fig. 3C). We sought to re-analyze the Ngô human dataset, hypothesizing that the presence of Ao in both RUB and COUGAR provides a plausible mechanism for an immune-mediated hypervirulence of *T. gondii*.

**Figure 7.**
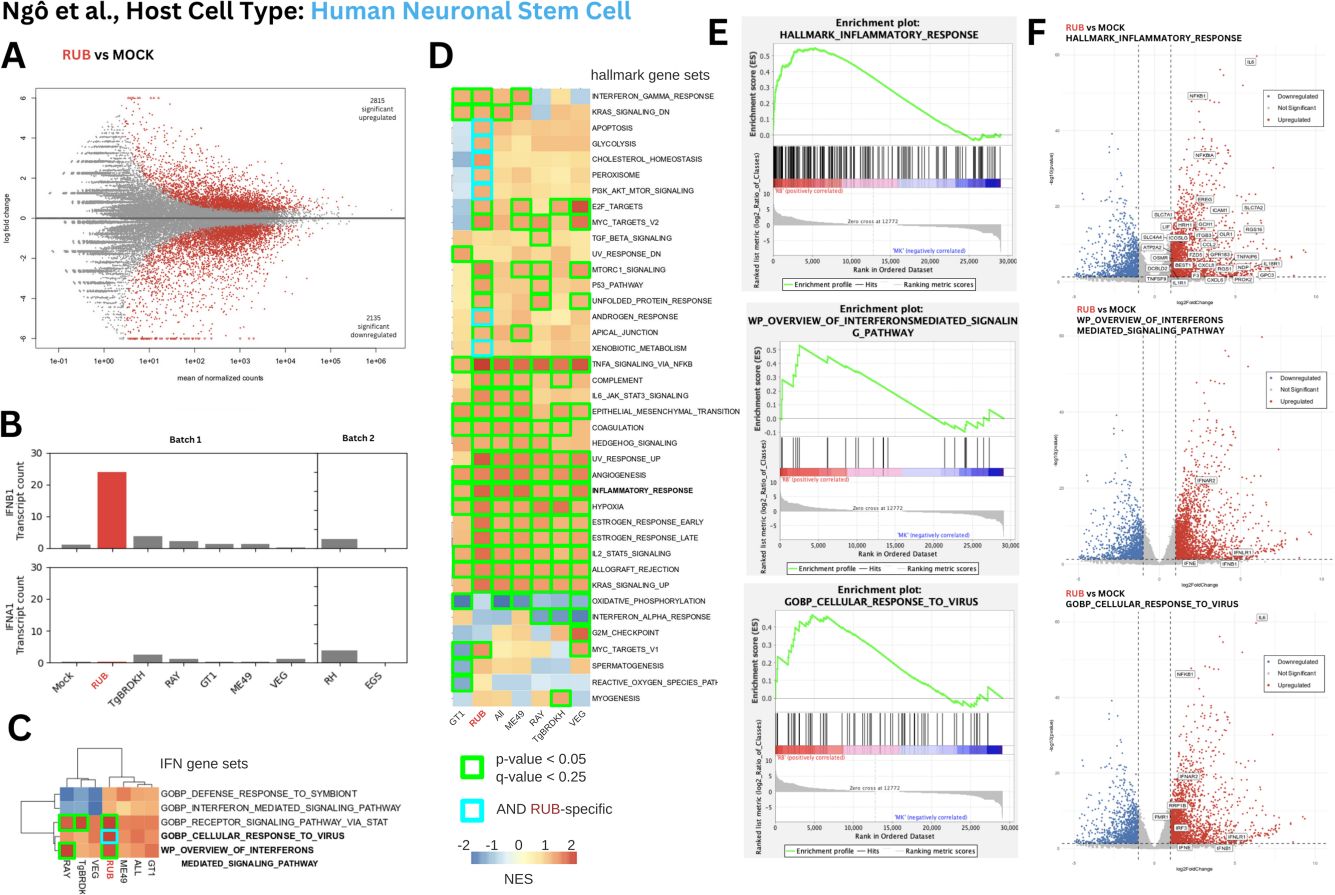
Differential gene expression (DGE) of human neuronal stem cells infected with various *T. gondii* strains. (A) MA plot of *T. gondii*—RUB vs Mock genes (highlighted: Benjamini-Hochberg adjusted *P*-value < 0.05). (B) Bar plot of normalized transcription counts of IFNB1 and IFNA1 across *T. gondii* strains and Mock sequence in Ngô *et al.*’s experiments, separated by batch. (C) Heat map of Normalized Enrichment Scores (NES) from GSEA using gene sets possessing interferon-specific genes, namely IFNA1 and IFNB1, applied to the *T. gondii* strains. (D) Heat map of NES values from GSEA using the Hallmark gene sets; only significant gene sets are shown. (E) GSEA curves comparing RUB vs Mock strain using gene sets of inflammatory response, cellular response to virus, and interferon-mediated signaling pathway. (F) Volcano plot of differentially regulated genes with significant members of notable gene sets being labeled.

Using Ngô *et al*. datasets, we performed differential gene expression (DGE) analysis and gene set enrichment analysis (GSEA) to test if the human immune response to Ao+ *T. gondii* strains was similar to the murine macrophage response from Melo *et al*., characterized by upregulation of the type I interferon gene *Ifnb1*. Ngô *et al*.’s *T. gondii*—RUB infection RNAseq data were available in two cell types, macrophage (*n* = 2) and neuronal stem cells (NSCs, *n* =2). We quantified human *IFNB1* induction in neuronal (Fig. 7) and macrophage cell types (Supplementary Fig. S7) across all *T. gondii* or mock datasets. We noted a large variation in *IFNB1* gene expression, especially across the macrophage datasets including a second set of mock controls, which could be explained by a sequencing batch-effect in the data (Supplementary Fig. S7 and Supplementary Table S1). Samples segregated by batch in a principal component analysis (Supplementary S8A) are indicative of global profile differences; thus, we limit DGE analysis to intra-batch comparisons.

Amongst the comparable *T. gondii*-infected neuronal samples, the RUB strain (*n* = 2) induced the highest *IFNB1* expression (26.1-fold increase vs Mock; *n* = 2, *P* = 0.029) relative to TgBRDKH (*n* = 2), GT1 (*n* = 2), ME49 (*n* = 2), RAY (*n* = 2), or VEG (*n* = 2) (Fig. 7B), in agreement with the murine *Ifnb1* expression in the Melo study (see also Fig. 3C). Next, we sought to contextualize the type of *IFNB1* response, asking which of the five gene sets containing *IFNB1*, if any, are differentially regulated in RUB versus mock and the rank of this difference amongst other *T. gondii* strains. While infection with both RUB and RAY strains shows upregulation of the gene signature for ‘Interferon mediated signaling pathway’ (*P*-value 0.009 and 0.049) and ‘Receptor signaling via STAT’ (*P*-value 0.006 and 0.039), a known downstream effector of interferon, the RUB strain was specifically enriched for ‘cellular response to virus’ (*P*-value 0.019), supporting the existence of specific host biological viral-response against Ao.

To characterize the broader host response of *T. gondii* RUB, we performed GSEA using the well-defined hallmark gene set (Liberzon et al. 2015). As expected, all *T. gondii* infections induce an ‘Inflammatory Response’ with the involvement of the ‘TNFA signaling via NF$\kappa $B’ pathway relative to mock controls. Yet the magnitude of these responses in neurons is the highest in RUB when compared to other (non-viral) strains (Fig. 7D). In addition, RUB was the only *T. gondii* strain inducing ‘Apoptosis’, ‘Glycolysis’, ‘Peroxisome’, and the ‘PI3K AKT MTOR Signaling’ axis, which altogether is consistent with the Melo study conclusions that *T. gondii* RUB is exceptionally pro-inflammatory, even amongst strains of *T. gondii*.

A similar inflammatory response trend is observed in the *T. gondii* RUB macrophage-infection experiments, but the response is less specific/exceptional (Supplementary Fig. S7). A higher overall level of experimental variation in the macrophages (*n* = 2 each) is evident when comparing the MA plots across cell types for statistically differentially regulated genes (compare NSC Fig. 7A and macrophages Supplementary S7A).

**Figure 8.**
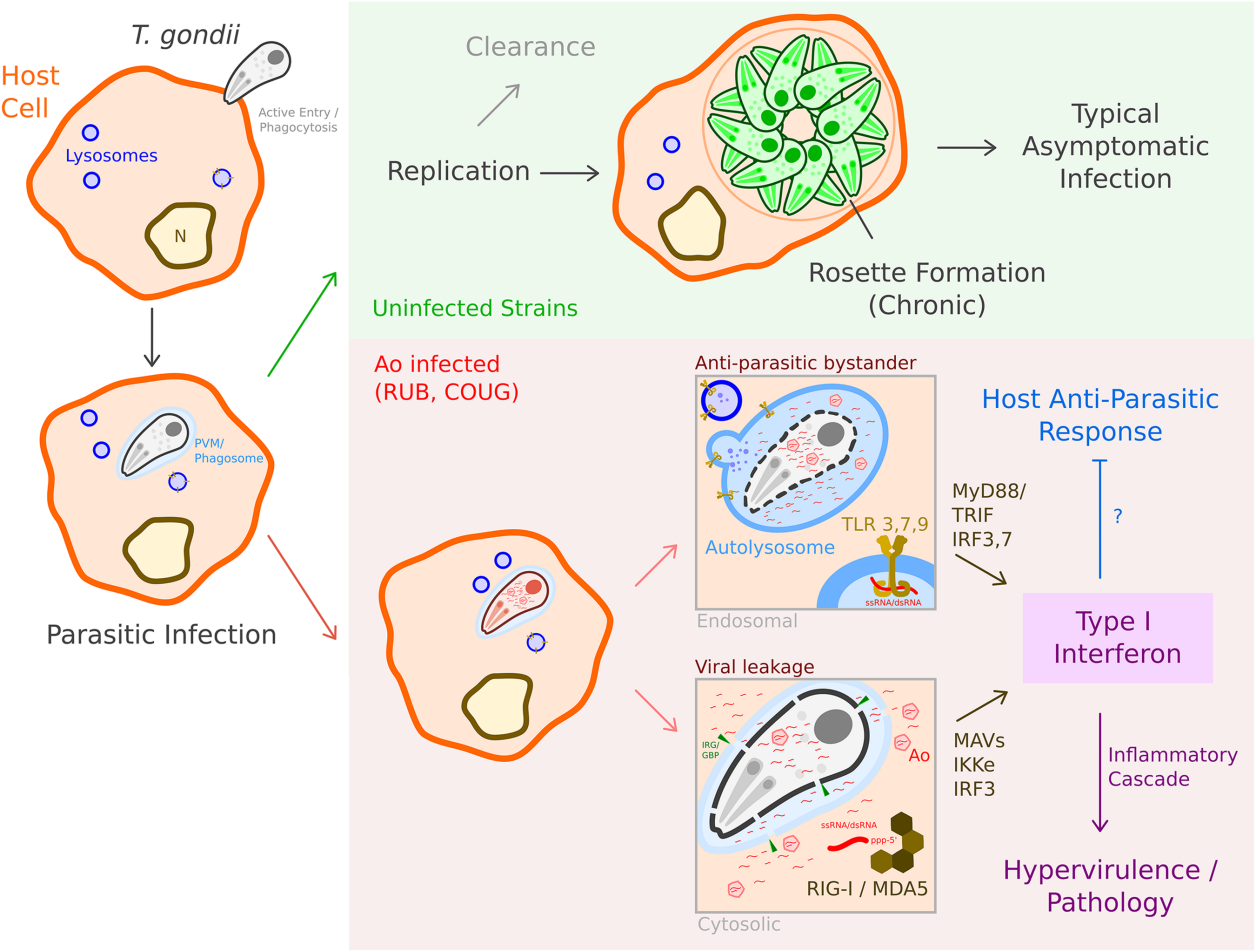
Hypervirulence model. *T. gondii* infects host cells. In uninfected strains, this either leads to successful clearance by the host or the establishment of chronic infection, typically asymptomatic. In virus+ strains, such as RUB and COUGAR, detection of viral RNA (vRNA)—either by endosomal TLRs after phagolysosome maturation following phagocytosis, or by RLRs surveying the cytosol after interferon-inducible GTPase (IRG/GBP)-mediated destruction of the parasitophorous vacuolar membrane—leads to the induction of type I IFN. The effect of this is twofold—the viral pathogen-associated molecular pattern drives additional inflammation which leads to pathology and overt symptoms, but potentially also impairs or diverts host anti-parasitic immunity. IRG: Immunity Related GTPase; GBP: Guanylate Binding Protein.

A major limitation of these *in silico* analyses is the impossibility of establishing a causal relationship between Ao and the host inflammatory responses. Notably, *T. gondii* RUB genetic factors are likely to confound this analysis. Yet we are able to recapitulate a statistically significant association of the Ao+ RUB strain with an inflammatory response in human cells. In the Melo study, this inflammatory response was experimentally demonstrated to be nucleic-acid mediated which, taken together with the discovery of Ao, allows us to tentatively propose a virus-mediated hypervirulence model (Fig. 8).

## Discussion

We discover a novel narnavirus, *Apocryptovirus odysseus*, which tightly associates with two distinct yet hypervirulent strains of *T. gondii*—RUB and COUGAR. Ao is a member of a broadly distributed clade (putatively at the genus level) of narnaviruses, the *Apocryptovirus*. We also describe eighteen additional novel species in *Apocryptovirus*, which likely infect apicomplexans, which in turn infect chordates. Furthermore, we provide initial (*in silico*) evidence to assert that Ao within *T. gondii* may act upon the innate immune system of human and mouse cells and therefore may be a hitherto uncharacterized hypervirulence factor for this ubiquitous parasite. While PPVs have been described for decades, their entanglement in the parasite–host relationship and pathogenesis is more recent (Gómez-Arreaza et al. 2017; Zhao et al. 2023). Multiple causal studies establish a molecular mechanism by which parasite-infecting viruses trigger chordate innate immune responses. Examples of this menage à trois include TVV in *Trichomonas vaginalis* (Fichorova et al. 2012), Leishmania RNA virus 1 (LRV1) in *Leishmania Viannia sp*. (Ives et al. 2011; Eren et al. 2016), GLV1 in *Giardia lamblia* (Pu et al. 2021), and CSpV1 in *Cryptosporidia sp*. (Deng et al. 2023). Unlike Ao, previously characterized PPVs are double-stranded RNA viruses, and a narnavirus contributing to disease in vertebrates has not yet been characterized. A recurrent theme amongst these studies is enhanced host pro-inflammatory cytokine production, specifically interferon, which can worsen parasite virulence.

Given the recurrent examples of PPV-mediated pathogenesis and the scarcity of examples of narnavirus infection-related phenotypes in the viral host, it is parsimonious to suggest that the presence of Ao RNA is a sufficient trigger of macrohost IFN-driven immune responses (Fig. 8). Melo et al. found that induction of type I IFN in the COUGAR strain is dependent upon the RNA-sensor RIG-I, and the signal cascade proceeds through MyD88/TRIF and ultimately IRF3, an inducer of type I IFN (Melo et al. 2013). IRF3 phosphorylation and IFN induction are also observed in other PPV infections, albeit with a double-stranded RNA ligand (Ives et al. 2011; Fichorova et al. 2012; Eren et al. 2016; Deng et al. 2023). Not knowing of the existence of Ao within their experiment, the authors ascribe COUGAR/RUB hypervirulence to parasite genomic DNA/RNA, potentially associated with the enhanced killing observed in human foreskin fibroblasts (HFFs), leading to release of greater quantities of *Toxoplasma* nucleic acids (Melo et al. 2013). This is plausible and not necessarily inconsistent with a contribution of vRNA to the innate response, although whether similar differences in killing can also be seen after phagocytosis by antigen-presenting cells such as macrophages (a fundamentally different process to infection of a fibroblast, during which the *Toxoplasma* mediates its own entry into a parasitophorous vacuole) is unclear. It also cannot explain the specificity of type I IFN response in these two strains and absence from the remaining twenty-nine (Ao- and ApoV-) strains which also possess parasite nucleic acids (Melo et al. 2013). With this in mind and in the absence of similar enhanced killing data for the RUB strain, we find that the presence of Ao in both strains remains the single most persuasive explanation for the differential host immune response. We also note that Melo et al. find significant heterogeneity between the transcriptomic profile of both murine macrophages responding to infection by these two strains and indeed between the two strains themselves. The only obvious transcriptomic similarity is the induced type I IFN response, which can be neatly explained by the key common denominator which separates the two otherwise unrelated COUGAR and RUB strains from all others—Ao positivity. We do note differences between existing pathways previously elucidated for innate response to PPVs and a potential contribution of Ao to pathogenicity. The other cases involve dsRNA viruses via TLR3/IRF3, whereas a ssRNA narnavirus genome would likely trigger TLR7,9 (ssRNA ligand) or a RIG-like receptor (uncapped RNA), and downstream of this, display some dependence on IRF7 (and, had it been investigated, IRF5) (Schoenemeyer et al. 2005; Jensen and Randrup Thomsen 2012). Melo et al. however found that murine macrophages infected by COUGAR strain tachyzoites *Irf7[KO]* in fact increases *Ifnb1* expression (Melo et al. 2013). This discrepancy could arise due to pleiotropic effects in constitutive immune gene knockouts, and will require additional experimental evidence to be resolved.

There is good evidence to suggest that the type I IFN response plays an important role in the control of *Toxoplasma*, even if debate continues around the direction of this response (i.e., whether type I IFN limits or supports infection). Knockout of *Ifnar* appears protective and supplementation of type I IFN detrimental after intraperitoneal injection of PRU strain *Toxoplasma* in mice (Hu et al. 2022). Similarly, an intestinal epithelial cell-specific knockout of *Ifnar* is protective in mice after oral infection of *Cryptosporidium* (another apicomplexan pathogen) carrying CSpV1 (Deng et al. 2023). In *Leishmania donovani* infection, *Ifnar*^-/-^ mice have significantly lower parasite burden compared to wild-type (Kumar et al. 2020). The authors propose that type I IFNs inhibit dendritic cell activity that would otherwise prime a type II IFN-driven, CD4^+^ Th1 cell-mediated anti-parasitic defence; they also demonstrate that ruxolitinib, a small molecule inhibitor of JAK kinases involved in interferon transduction, exerts anti-parasitic effects that are not observed in *Ifnar^-/-^* mice, suggesting a dominant effect via inhibition of type I IFN signaling (Kumar et al. 2020).

On the other hand, others have found that after oral infection of ME49 cysts, *Ifnar^-/-^* mice had significantly poorer survival rates and increased numbers of brain cysts (Matta et al. 2019). Differences between strains, route of infection, and multiplicity of infection, with effects on the timing, duration, and magnitude of the type I IFN signal, could explain these contrasting findings (Silva-Barrios and Stäger 2017; Lee and Ashkar 2018). Regardless of the eventual outcomes on parasite burden and host survival, we propose that the additional type I IFN response, brought on by the presence of Ao vRNA, will at least acutely contribute to more severe disease in COUGAR and RUB strain infection. While it has been suggested that PPVs may promote pathology driven by their host via immune modulation of the macrohost, such as has been recently observed in *Cryptosporidium* (Deng et al. 2023), we do not expect this to hold true across all parasite strains, species, and hosts; virus–parasite–host fitness relationships are by definition complex.

If it is indeed the case that PPVs cause exacerbated immune response in parasitic infection, it may be worthwhile to investigate antivirals as a form of treatment. This has already been suggested in the case of TVV+ *T. vaginalis*, wherein the canonical anti-parasitic, metronidazole, can counterintuitively exacerbate inflammatory signaling in infected epithelia (Fichorova et al. 2012; Narayanasamy et al. 2022). Elimination of LRV1 by RdRp inhibition resulted in the generation of strains which induce weaker inflammatory responses and were associated with reduced pathology *in vivo* (Kuhlmann et al. 2017). Similar investigations have focused on the efficacy of antivirals indinavir and ribavirin, individually or in combination with the anti-parasitic paromomycin against CSpV1+ *Cryptosporidium* in a 2D monolayer model of human enteric epithelia (Deng et al. 2023). Efforts during the COVID-19 pandemic highlighted RdRp as an important therapeutic target in RNA virus antiviral therapy (Tian et al. 2021). Given the potential dependence of the efficacy of such drugs on RdRp structure, further elucidation of the structure in the *Narnaviridae* and proximal clades, using conventional wet lab approaches and molecular docking software, may build on our observations of conserved residues in Ao and related viruses to support the identification of effective antivirals. Besides, the generation of virus-free parasite strains will allow for elucidation of the contribution of endogenous virus to host and macrohost physiology (Kuhlmann et al. 2017; Espino-Vázquez et al. 2020; Narayanasamy et al. 2022; Kuroki et al. 2023).

While *T. gondii* pathogenicity is certainly multi-variate, with host immune status, age, and parasite genetic variation known influences on pathophysiology, there is still a lack of reliable prognostic indicators. A viral hypervirulence factor can explain, at least in part, some of the stochastic nature of parasitic disease burden in humans and other animals. Given the enormous infectious burden of *T. gondii* and other parasites (impacting multiple billions of people), it is prudent to improve monitoring for viruses within the niche of the human biome, as it interlinks human health with the health of microbial flora. In general, if the presence of PPV is prognostic (as it is in TTV/CSpV1), then ApoV± status can impact treatment and outbreak-management decisions in toxoplasmosis and other parasitic diseases.

Hypervirulence in *Toxoplasma* can present with different symptoms. Broadly, however, we note that in humans, both infections with COUGAR or RUB are associated with ocular involvement and overt acute pathology even in immunocompetent patients (Bowie et al. 1997; Dubey 2021; Dardé et al. 1998). In mice, both strains are associated with increased mortality (Khan et al. 2009; Niedelman et al. 2012; Hassan et al. 2019). COUGAR is associated with enhanced oral infectivity when compared to other strains, although whether RUB shares this phenotype is unknown (Su et al. 2003). A recent report of COUGAR strain infection in four southern sea otters (*Enhydra lutris nereis*) observed severe myocarditis and marked subcutaneous and peritoneal steatitis (Miller et al. 2023). Type I IFN signaling is thought to play an important role in driving adipocyte inflammation (Chan et al. 2020), so steatitis could present an unusual consequence of the vRNA contribution to toxoplasmosis in Ao+ strains. Ultimately, these proposed mechanisms and models require experimental validation, for which we outline an efficient set of critical experiments. First, necessity and sufficiency of Ao-mediated IFN production/pathogenicity needs to be established. For necessity, Ao infection of a naive *T. gondii* strain (ME49) will promote *IFNB1* expression relative to an uninfected control. Curing of COUGAR/RUB *T. gondii* of the virus (e.g. by serial passage in antiviral-supplemented culture until Ao- status can be confirmed by PCR) will cause a loss of IFN production/pathogenicity (sufficiency), and this effect can be rescued by subsequent re-infection of the cured strain. The gain and loss of type I IFN responses will be dependent on ssRNA receptors TLR7,9 or RLRs, and not TLR3 or TLR11,12. These critical experiments, when performed *in vitro*, would establish a causal relationship between Ao and proinflammatory induction, while the same set of experiments in an animal model can formalize the relationship between Ao and parasite pathogenicity.

The objective of this study was to rapidly screen for a candidate highly divergent neuroinflammatory virus *in silico*. Here we discovered *Apocryptovirus odysseus*, a narnavirus likely infecting *T. gondii*, which, through the innate immune sensing of vRNA, could drive a type I IFN-mediated inflammatory response. While we cannot establish this mechanism causally, it is plausible and supported by the body of available mechanistic and transcriptomic evidence.

A link between neurotropic toxoplasmosis and neuroinflammatory and neurodegenerative conditions, such as Alzheimer’s disease (AD), has been proposed (Nimgaonkar et al. 2016; Nayeri Chegeni et al. 2019; Tyebji et al. 2019; Yang et al. 2021). More recently, more mechanistic studies have been able to link *Toxoplasma* infection of the central nervous system (CNS) with several correlates of AD pathology—disruption of the blood-brain barrier, glial activation and synapse loss, *inter alia* (Li et al. 2019; Ortiz-Guerrero et al. 2020; Castaño Barrios et al. 2021; Anaya-Martínez et al. 2023; Carrillo et al. 2023). The picture however remains far from conclusive. Other epidemiological works have found no significant effect of *T. gondii* seropositivity on cognitive function in adult patient populations (Wyman et al. 2017; Torniainen-Holm et al. 2019). In pre-clinical models, evidence has been provided for neuroprotective effects in AD mouse models (Jung et al. 2012; Cabral et al. 2017), while others still find no significant effect on murine age-related cognitive decline (McGovern et al. 2020). This underscores the importance of a better understanding of factors which influence *Toxoplasma* pathogenicity, which could explain why some patients develop CNS pathology, while others are seemingly unaffected, and even protected. In AD, CNS inflammation is balanced between beneficial (e.g. priming microglia for amyloid plaque clearance) and detrimental (e.g. synaptic loss, immune infiltration) functions (Heneka et al. 2015; Leng and Edison 2021). *Toxoplasma* strain-dependent effects may go a long way toward clearing up the somewhat mixed picture observed to date (Cabral et al. 2017; Xiao, Savonenko, and Yolken 2022). The presence of an *Apocryptovirus*, or similar PPV, is well placed to skew the inflammatory equilibrium in chronic toxoplasmosis toward neuropathology, particularly when one considers the recent identification of a detrimental role for type I IFN signaling in AD (Roy et al. 2020, 2022; Sanford et al. 2023).

The strength of computational virology is in its capacity for searching massive swathes of data and identifying novel and unexpected relationships between viruses and diseases. As the number of newly discovered viruses continues to grow, analysis for developing specific and evidence-based hypotheses can help focus where resource-intensive biological experiments should be allocated. Ao and neuroinflammation is such a case study, and serves to demonstrate the need for, and merit of, the comprehensive characterization of Earth’s RNA virome.

## Materials and methods

### Querying the BioSample database

In the initial screen for novel and highly divergent neurotropic viruses, we searched the BioSamples SQL table in the serratus SQL database (https://github.com/ababaian/serratus/wiki/SQL-Schema) with the query SELECT * FROM tismap_summary WHERE biosample_tags LIKE ‘%neuron%’ AND scientific_name $ = $ ‘Homo sapiens’ AND percent_identity $ < $90 ORDER BY coverage desc; and identified SRA run SRR1205923 in BioProject PRJNA241125 through manual curation. blastp and blastx (Altschul et al. 1990) were performed with a query of the u150420 palmprint identified in three separate libraries to measure the percent amino acid identity to known viral proteins using the non-redundant proteins database (nr). Furthermore, blastn against the nr/nt database was performed (date accessed: 9 June 2023).

### Viral genome identification, assembly, and endogenous virus evaluation

The three libraries (SRR1205923, SRR1204654, and SRR1205190) identified through a serratus SQL search were *de novo* assembled using rnaspades 3.15.5 (Bushmanova et al. 2019). rnaspades was run with parameters ‘–rna –s1 -t 64’. palmscan (version 2, –threads 64 –seqtype nt) (Babaian and Edgar 2022)—an RNA-dependent RNA Polymerase detector—was used to identify the viral RdRp contig in the assembled transcript file. For each library, the contigs identified by palmscan were re-analyzed through orffinder (NCBI RRID:SCR_016643) and the longest ORF was extracted. This amino acid sequence was identical across both mRNA libraries SRR1205923 and SRR1204654, but contig in SRR1205923 had a low-coverage insertion which introduced a frameshift mutation. This was manually corrected to the consensus sequence to result in an identical coding sequence to the other libraries. To test if the ORF encoded a plausible RdRp, the structure was predicted with colabfold (AlphaFold_mmseq2, v1.5.5) (Mirdita et al. 2022), using the putative RdRp ORF from SRR1205923. bwa-mem 0.7.17 -t 64 (Li and Durbin 2010) was used for aligning SRR1205923 transcripts generated by the assembler back to SRR1205923’s reads (FASTQ); alignment was visualized in igv 2.16.0 (Robinson et al. 2011). The *T. gondii* RUB and *T. gondii* COUGAR DNA-seq data were downloaded from their respective BioProjects (PRJNA61119 and PRJNA71479). bowtie2 (version 2.5.1, ‘–local –very-sensitive-local –threads 64 -q -x -U’) (Langmead and Salzberg 2012) was used to map the DNA-seq reads to a reference database of Ao contig 1 and 2, and no reads were alignable.

### Identification of Ao contig 2 via host read-depletion

Reference genomes for human (GRCh38, accessed from NCBI (https://www.ncbi.nlm.nih.gov/datasets/genome/GCF_000001405.26/) and *Toxoplasma gondii* ME49 (TgondiiME49, accessed from ToxoDB (Gajria et al. 2008)) were downloaded and concatenated into one reference dataset (hgTg). Assembled contigs (rnaspades) for each of the four RUB mRNA Ngô libraries (SRR1205923, SRR1204654, SRR1204653, and SRR1204652) were aligned to hgTg with bowtie2 (version 2.5.1, ‘–local –very-sensitive-local –threads 64 -f -x -U’). samtools 1.17 view with parameters ‘-S -b -@64’ converted the SAM to a BAM file-format and another samtools view command with parameters ‘-f 4 -b -@ 64’ removed mapped contigs, retaining non-human, non-*Toxoplasma* mapping contigs. The four libraries (SRR1205923, SRR1204654, SRR1204653, and SRR1204652) had 76, 92, 52, and 50 unmapped contigs, respectively. All unmapped contigs were converted to FASTA with samtools and were then aligned against NCBI’s nr proteins database using diamond 2.1.8 (Buchfink, Xie, and Huson 2015) in blastx mode with parameters ‘–masking 0 –unal 1 –mid-sensitive -l 1 —p14 —k1 –threads 64 —f 6 qseqid qstart qend qlen qstrand sseqid sstart send slen pident evalue full_qseq staxids sscinames’. The contig with the highest coverage matched *E. coli* sequence, the third highest by coverage was the Ao RdRp contig and the second highest across all four RUB libraries, was a $ \approx 1280$ nucleotide long contig with no known associations through blast. This unknown contig was common throughout all four RUB libraries and was called the putative contig 2. Subsequently, we show this contig correlated with contig 1 (RdRp) across the Ngo BioProject, and we identified co-evolving contig 2 homologs in nineteen related *Apocryptovirus sp*., supporting the fact that contig 2 is viral in origin. Subsequently, a genome map representation was created for both contig 1 and contig 2 with gggenes 0.4.1 (Wilkins 2020), an r 4.2.2 package for drawing gene arrow maps and ggplot2 3.4.0 (Wickham 2016). Finally, a Pearson correlation coefficient was calculated with ggpubr 0.6.0 in r (Kassambara 2023).

### Assembling and quantifying of Ngô *et al.* libraries

A total of 117 out of the 237 libraries in the Ngô et al. (2017) BioProject are labeled as mRNAseq data, the other 120 libraries are micro RNA (miRNA). The 117 mRNAseq libraries were pre-fetched and the FASTQ files were extracted using sratoolkit 3.0.5. Next, all 117 Ngô mRNA libraries were *de novo* assembled with rnaspades with parameters ‘–rna—s1—t 64’. Using bowtie2 ‘-build’, a dataset was created with all four genomes/sequences: Human (HgCh38), *T. gondii* ME49 (Tg64), Ao contig 1 RdRp, and Ao contig 2. Next, the reads for all 237 libraries in the Ngô *study* were aligned against this dataset using bowtie2 with parameters ‘–local –very-sensitive-local –threads 64 —q —x —U’. To convert the SAM file to a BAM file, samtools with parameters ‘-S—b—@ 64—F 260’ were used. Flag—F 260 removes all unmapped reads and non-primary alignments. The output of samtools was then piped into seqkit 2.4.0 (Shen et al. 2016) with the ‘-c’ parameter which counts the mapped reads per chromosome/sequence.

### Assembling and quantifying Melo *et al.* libraries

Similarly to the approach taken with libraries from Ngô *et al*., the FASTQ files for all thirty-two libraries were downloaded using sratoolkit for the BioProject from Melo et al. (2013). All thirty-two Melo libraries were *de novo* assembled with rnaspades parameters ‘–rna –pe —t 64’. bowtie2 was used to create a dataset with all four genomes/sequences: Murine (GRCm39), Tg64, Ao contig 1 RdRp, and Ao contig 2. Next, the reads for all thirty-two libraries by Melo et al. were aligned against this dataset using bowtie2. To convert the SAM file to a BAM file, samtools was used (see ‘Assembling and quantifying of Ngô et al.’s libraries’ for parameters). All the metadata for the entire Melo *et al*.’s BioProject (PRJNA241125) was collected with Google’s bigquery tool. Using this metadata and the data parsed with pandas 2.0.3 (team, The pandas development 2020), 3 was generated in r with dplyr and ggplot2.

### Screening for and assembling the apocryptoviruses

To identify RdRp related to Ao, we aligned the sOTU in palmDB (version 2023-04) (Babaian and Edgar 2022) to the Ao palmprint (usearch -usearch_global palmDB.palmprint.faa -db ao.palmprint.faa -id 0.3), yielding matches in ‘u380516’, ‘u476932’, ‘u706419’, ‘u492272’, ‘u602981’, ‘u1051092’, ‘u845773’, ‘u857620’, ‘u665910’, ‘u819619’, ‘u584295’, ‘u691934’, ‘u71279’, ‘u145522’, ‘u626963’, ‘u150420’, ‘u592253’, ‘u828652’, ‘u617666’, ‘u964003’, ‘u643849’, ‘u993146’, ‘u770301’, ‘u533578’, ‘u460145’, ‘u419915’, ‘u849389’, ‘u1004674’, ‘u942391’, ‘u10732’, ‘u761722’, ‘u599206’, and ‘u1009116’. To query which serratus-processed SRA runs contained microcontigs matching these palmprints, we performed the SQL query ‘SELECT * FROM palm_sra2 WHERE qc_pass = ‘true’ AND sotu IN (‘u380516’, ‘u476932’, ‘u706419’, ‘u492272’, ‘u602981’, ‘u1051092’, ‘u845773’, ‘u857620’, ‘u665910’, ‘u819619’, ‘u584295’, ‘u691934’, ‘u71279’, ‘u145522’, ‘u626963’, ‘u150420’, ‘u592253’, ‘u828652’, ‘u617666’, ‘u964003’, ‘u643849’, ‘u993146’, ‘u770301’, ‘u533578’, ‘u460145’, ‘u419915’, ‘u849389’, ‘u1004674’, ‘u942391’, ‘u10732’, ‘u761722’, ‘u599206’, and ‘u1009116’), returning the 185 matching libraries (Supplementary Table S3). Sorting by microcontig coverage for each palmprint-library, FASTQ files from the top three libraries were downloaded using sratoolkit and assembled using rnaspades with parameters ‘–rna —t 64 –pe’. RdRp-contigs were identified as described above in each library.

### MSA and phylogenetic tree of Ao and related narnaviruses

In order to sample Ao-related viruses, we first searched for available sequences in GenBank’s non-redundant protein (nr) database, using psi-blast and default parameters with the BLOSUM62 substitution matrix (date accessed: June 2023). We queried the database with the entire amino acid sequence for the Ao RdRp recovered from the RUB strain of *T. gondii* (SRR1205923); all resulting sequences had E-values $ < 7e - 28$. Notably, this approach failed to capture more distantly related narnaviruses, such as those previously described in arthropod metagenomic samples, yeast, and nematodes. Thus, we supplemented the MSA with RdRps from an additional blastp search on *Coquillettidia venezuelensis* Narnavirus 1 (QBA55488.1). For a more distant clade, we selected seven ICTV-recognized *Mitoviridae* species, two from each genus except *Kvaramitovirus*, for which there is only one accepted species. As an outgroup, we added two species from the family *Fiersviridae*: the bacteriophages MS2 and Qbeta. The sequence with the highest percent identity relative to Ao RUB among these was QIM73983.1 at 53.53 per cent identity, but only 40 per cent coverage. For this reason, after aligning the sequence with muscle v3.8.1551 (Edgar 2004), we decided to manually trim the MSA to the RdRp palm (motifs F-E) and thumb subdomains. This ensured partial contigs could be fairly represented in the phylogeny. Some blast hits, however, were too incomplete to satisfy this requirement and were therefore removed. With the closest relative in GenBank at such a low identity, we re-queried the SRA for Ao-like sequences. Using diamond, a sequence database of all Ao-like palmprint sOTUs was created. We then aligned all assembled transcripts from Ao-like RdRp-containing libraries to the Ao-like palmprints (diamond, ‘-blastx –very-sensitive –threads 64 —f 6 qseqid sseqid pident length mismatch gapopen qstart qend sstart send evalue bitscore full_qseq’). The alignment outputs were merged and transcripts with 100 per cent identity to the palmprint, $ \approx 3000$ nt length, and the highest coverage if multiple choices were available were chosen as the representative contigs for each virus. A total of eight of the fifty-five libraries did not have a high-quality representative contig, and were discarded. The remaining contigs were analyzed with orffinder command line to find the ORFs. The longest ORF was chosen, and then confirmed to be the RdRp using blastp. These RdRp ORFs were combined into one FASTA file, and are the source of Ao-like virus RdRp. The final MSA contains 169 RdRps; a full list of the selected sequences, their GenBank/SRA accessions, and associated metadata is available in the Supplementary Materials. Based on the RdRp palm, we generated a maximum-likelihood phylogenetic tree using iq-tree v2.2.2.7 (Minh et al. 2020) and viewed in ggtree v3.10.0 (Yu et al. 2016), where the substitution matrix was selected as rtREV + F + I + R8 by modelfinder (Kalyaanamoorthy et al. 2017). Bootstrap values on 1,000 replicates were generated via ufboot v1 (Hoang et al. 2018).

### Novel Ao-proximal RdRp Motif identification

Based on the phylogeny, sequences displaying high-relatedness with Ao were selected. The full sequences (as opposed to just the palm and thumb) were retrieved and aligned with muscle. A conservation map showing alignments for selected sequences in the MSA was generated using ggmsa v1.8.0 (Zhou et al., 2022a). Subsequences with high occupancy and conservation were selected as novel motifs $\alpha $–$\mu $. We also selected ‘non-motif’ subsequences to serve as a background. These constituted the high occupancy regions between motifs. Sequence logos for the novel and canonical RdRp motifs were generated via weblogo v2.8.2 (Crooks et al. 2004). Pairwise conservation was calculated based on the percentage of residues with similar chemical properties between two aligned subsequences. No penalties were given to mismatched residues or gaps. A matrix of pairwise comparisons between all motifs across all selected sequences was calculated with pandas. All FASTA files and the Jupyter notebook used for calculation are available in the supplementary data. Mean conservation across the novel, core, and non-motifs was calculated and used as the basis for a scatterplot in r using ggplot2. Deltas (differences between mean values for the novel, core, and non-motif sets) were also plotted with ggplot2 in the form of a violin plot.

### RdRp structure analysis

Protein structures of full-length RdRps were predicted using colabfold for the Apocryptoviruses and select Apocrypto-proximal RdRps. Structures were visualized in PyMol (Schrödinger 2015). The topology map was generated with pdbsum (10 April 2023 version) (Laskowski et al. 2018) and further edited with adobe illustrator. tm-align v20170708 (Zhang and Skolnick 2005) was used for structural alignments, and the colorbyrmsd plugin (6 April 2016 version) for PyMol was used to generate the distance maps.

### Contig 2 recovery

To recover contig 2 for each Ao-like virus, a diamond database of all four mRNA RUB Ngô Ao contig 2s was created. Using blastx, the transcripts for all Ao-like viruses were aligned against this database. pandas was used to sort and combine all the alignment data. For each library, the contig with the best alignment identity, e-value, coverage, and length was chosen. These contigs were combined into one FASTA file. This combined file was then put through the orffinder command line and all the ORFs were extracted. An MSA of the extracted ORFs was created using muscle. Upon viewing the alignment, two profiles were generated: one for contig 2’s pORF1 and the other for ORF2. Based on these profiles, two HMM models (one for ORF 1 and one for ORF 2) were created with hmmer3 v3.4. Using these HMM models, hmmer’s hmmsearch was used to search all Ao-like virus libraries. Any ORFs that hit against the HMM model were labeled either pORF1 or pORF2 according to which HMM model they aligned against.

### DGE

To begin, the human reference genome (GRCh38.p14), its corresponding gene annotation, as well as the FASTQ files of the Ngô dataset accessions for Macrophage (MonoMac6) and Neuronal Stem Cell (NSC) were downloaded. Initially, all experiments were performed on macrophages, and then replicated with the NSC dataset. hisat2 v.2.21 (Kim et al. 2019) was used to extract splice sites and exons, and then used to index the human genome to generate SAM files for each accession. SAM files were converted to BAM files using samtools (see ‘Assembling and quantifying of Ngô et al.’s libraries’). A matrix of counts per human gene symbol was generated using the featureCounts tool in the subread v.2.14.2 package (Liao, Smyth, and Shi 2014). deseq2 v.1.40.2 (Love, Huber, and Anders 2014) was used to analyze the count matrix and test for DGE. deseq2 and ggplot2 were used to create MA plots, PCA plots, and volcano plots with highlighted values having Benjamini-Hochberg adjusted *P*-value $ < 0.05$. Upon further analysis, it was noted that of the four control mock strains used, two appeared to be pro-inflammatory (Supplementary Fig. S8). To investigate this, a spreadsheet consisting of all of the metadata from the Ngô study was created. From this, it was observed that Ngô et al. (2017) performed their experiments in separate runs. This batch effect explains the variation in the immune system response for the control Mock strains. To account for this batch effect, any samples that were used in batches that presented hyper-inflammatory symptoms were not used for further analysis. To determine whether the defined set of genes showed statistically significant differences between phenotypes, we used the Gene Set Enrichment Analysis (gsea v.4.3.2) software (Subramanian et al. 2005). Using the Molecular Signatures Database (MsigDB) (Liberzon et al. 2015), we focused on the hallmark collection of gene sets, as well as five non-hallmark gene sets. The five non-hallmark gene sets were chosen on the basis of containing the genes *IFNB1, IFNW1, IFNA13, IFNA1, IFNE, IFNL3, IFNL4*, interferon genes which have been observed to be upregulated in *T. gondii* infection. Using all of these gene sets, GSEA data matrices were then used to create both enrichment plots and heat maps of NES scores.

### Quantifying co-occurrence of *Apicomplexa* presence with apocryptoviruses

We selected the six non-laboratory source organisms in which apocryptoviruses were identified. Each of these sets was further partitioned into a contigency table of four groups according to two categorical variables. The first variable was whether or not there is significant apicomplexan signal ($ > 128$ matching reads) within that SRA run, as reported by stat under the phylum *Apicomplexa* (BigQuery: SELECT * FROM nih-sra-datastore.sra_tax_analysis_tool.tax_analysis WHERE tax_id = 5794; data were retrieved on July, 2023.). The second variable was whether or not the SRA run belongs to the proposed list of identified Apocryptoviruses (Supplementary Table S3). To test if these variables were associated, we performed a Fisher’s Exact Test (scipy.stats.fisher_exact); results are summarized in Fig. 5.

### Reverse transcriptase PCR for *A. odysseus* in cultured *T. gondii*

HFFs were cultured in Dulbecco’s Modified Eagle Medium from (Invitrogen, cat# 11965-118) supplemented with 10 per cent heat-inactivated fetal bovine serum (FBS, Genesee Scientific, cat# 25-550), 2 mM L-glutamine, and 50 µg/ml of both penicillin and streptomycin, along with 20 µg/ml gentamicin. *Toxoplasma* strains RH (Ao—control), RUB (Ao+), and COUGAR (Ao+) from Melo et al. (2013) were propagated in HFFs in T25 culture flasks. RNA was extracted using the RNeasy Plus Mini Kit (Qiagen, cat# 74134), following the manufacturer’s instructions. The yield and purity of the extracted RNA were assessed using a Nanodrop. cDNA was synthesized using the Reliance Select cDNA Synthesis Kit (Bio-Rad, cat#12012802) for 20 min at 50°C. Amplification of *A. odysseus* RUB/COUGAR was performed using primers: Forward Primer—5ʹ-ATTGTTCCCGTGCATGACTG-3ʹ and Reverse Primer—5ʹ-TCTTGAGAGTCCGGCTTTGG-3ʹ. As a loading and positive control for cDNA quality, *Toxoplasma GRA1* amplification was performed with primers: Forward Primer—5ʹ-TATTGTCGGAGCTGCTGCATCG-3ʹ and Reverse Primer—5ʹ-GCTCACTGCATCTTCCAGTTGC-3ʹ. To test if Ao is a potential endogenous viral element, the same reaction without reverse transcriptase was performed for all samples. PCR reactions with cDNA from RH, RUB, and COUGAR, and a no-template negative control were also included. The PCR was performed using MangoMix with MangoTaq (Bioline, cat# C755G90), for thirty-four cycles of denaturion (15 s at 95°C), annealing (30 s at 61°C (*A. odysseus*) or 59°C (GRA1)), and extension (30 s at 72°C). Amplicons were purified using the DNA Clean and Concentrator kit (Zymo Research, cat# 11-305C) and sequenced by QuintaraBio (3563 Investment Blvd, Suite 2, Hayward, CA 94545) with aforementioned primers. The *A. odysseus* RUB and COUG sequences were a 100 per cent match to *A. odysseus*_SRR446933 and *A. odysseus*_SRR446909, respectively.

## Supplementary Material

veae040_Supp

## Data Availability

*Apocryptovirus* sequences are available in GenBank under BioProject PRJEB71349. All sequences and supporting data are available at https://github.com/ababaian/serratus/wiki/Apocryptovirus. Project notebooks and code are available via S3 (https://mtnemo.s3.amazonaws.com/README.md).

## References

[R1] Abbo S. R. et al. (2023) ‘The Virome of the Invasive Asian Bush Mosquito Aedes Japonicus in Europe’, *Virus Evolution*, 9: vead041 . 10.1093/ve/vead041 PMC10460169 37636319

[R2] Ajioka J. W. et al. (1998) ‘Gene Discovery by EST Sequencing in Toxoplasma Gondii Reveals Sequences Restricted to the Apicomplexa’, *Genome Research*, 8: 18–28. 9445484 10.1101/gr.8.1.18

[R3] Altschul S. F. et al. (1990) ‘Basic Local Alignment Search Tool’, *Journal of Molecular Biology*, 215: 403–10. 2231712 10.1016/S0022-2836(05)80360-2

[R4] Anaya-Martínez V. et al. (2023) ‘Changes in the Proliferation of the Neural Progenitor Cells of Adult Mice Chronically Infected with Toxoplasma Gondii’, *Microorganisms*, 11(11): 2671. 10.3390/microorganisms11112671 PMC10673519 38004683

[R5] Anthony S. J. et al. (2013) ‘A Strategy to Estimate Unknown Viral Diversity in Mammals’, *MBio*, 4: e00598–13. 24003179 10.1128/mBio.00598-13 PMC3760253

[R6] Aramini J. J., Stephen C., and Dubey J. P. (1998) ‘Toxoplasma Gondii in Vancouver Island Cougars (Felis Concolor Vancouverensis): Serology and Oocyst Shedding’, *The Journal of Parasitology*, 84: 438–40. 9576522

[R7] Babaian A., and Edgar R. (2022) ‘Ribovirus Classification by a Polymerase Barcode Sequence’, *PeerJ*, 10: e14055. 10.7717/peerj.14055 PMC9573346 36258794

[R8] Barrett T. et al. (2012) ‘BioProject and BioSample Databases at NCBI: Facilitating Capture and Organization of Metadata’, *Nucleic Acids Research*, 40: D57–63. 22139929 10.1093/nar/gkr1163 PMC3245069

[R9] Batson J. et al. (2021) ‘Single Mosquito Metatranscriptomics Identifies Vectors, Emerging Pathogens and Reservoirs in One Assay’, *Elife*, 10: e68353. 10.7554/eLife.68353 PMC8110308 33904402

[R10] Bertranpetit E. et al. (2017) ‘Phylogeography of Toxoplasma Gondii Points to a South American Origin’, *Infection Genetics & Evolution*, 48: 150–5. 10.1016/j.meegid.2016.12.020 28028000

[R11] Bisetegn H. et al. (2023) ‘Global Seroprevalence of Toxoplasma Gondii Infection among Patients with Mental and Neurological Disorders: A Systematic Review and Meta-analysis’, *Health Science Reports*, 6: e1319. 10.1002/hsr2.1319 PMC10242185 37287705

[R12] Bittner N. K. J., Mack K. L., and Nachman M. W. (2022) ‘Shared Patterns of Gene Expression and Protein Evolution Associated with Adaptation to Desert Environments in Rodents’, *Genome Biology and Evolution*, 14: evac155. 10.1093/gbe/evac155 PMC9648513 36268582

[R13] Bjornevik K. et al. (2022) ‘Longitudinal Analysis Reveals High Prevalence of Epstein-Barr Virus Associated with Multiple Sclerosis’, *Science*, 375: 296–301. 35025605 10.1126/science.abj8222

[R14] Blake D. P., and Tomley F. M. (2014) ‘Securing Poultry Production from the Ever-Present Eimeria Challenge’, *Trends in Parasitology*, 30: 12–9. 24238797 10.1016/j.pt.2013.10.003

[R15] Bowie W. R. et al. (1997) ‘Outbreak of Toxoplasmosis Associated with Municipal Drinking Water. The BC Toxoplasma Investigation Team’, *The Lancet*, 350: 173–7. 10.1016/s0140-6736(96)11105-3 9250185

[R16] Buchfink B., Xie C., and Huson D. H. (2015) ‘Fast and Sensitive Protein Alignment Using DIAMOND’, *Nature Methods*, 12: 59–60. 25402007 10.1038/nmeth.3176

[R17] Bushmanova E. et al. (2019) ‘rnaSPAdes: A de Novo Transcriptome Assembler and Its Application to RNA-Seq Data’, *GigaScience*, 8: giz100. 10.1093/gigascience/giz100 PMC6736328 31494669

[R18] Cabral C. M. et al. (2017) ‘Dissecting Amyloid Beta Deposition Using Distinct Strains of the Neurotropic Parasite Toxoplasma Gondii as a Novel Tool’, *ASN Neuro*, 9: 1759091417724915. 10.1177/1759091417724915 PMC5565021 28817954

[R19] Carrillo G. L. et al. (2023) ‘Complement-dependent Loss of Inhibitory Synapses on Pyramidal Neurons following Toxoplasma Gondii Infection’, *Journal of Neurochemistry*. 10.1111/jnc.15770 PMC10363253 36683435

[R20] Castaño Barrios L. et al. (2021) ‘Behavioral Alterations in Long-Term Toxoplasma Gondii Infection of C57BL/6 Mice are Associated with Neuroinflammation and Disruption of the Blood Brain Barrier’, *PLoS One*, 16: e0258199. 10.1371/journal.pone.0258199 PMC8491889 34610039

[R21] Chan C. C. et al. (2020) ‘Type I Interferon Sensing Unlocks Dormant Adipocyte Inflammatory Potential’, *Nature Communications*, 11: 2745. 10.1038/s41467-020-16571-4 PMC7265526 32488081

[R22] Charon J. et al. (2019) ‘Novel RNA Viruses Associated with Plasmodium Vivax in Human Malaria and Leucocytozoon Parasites in Avian Disease’, *PLOS Pathogens*, 15: e1008216. 10.1371/journal.ppat.1008216 PMC6953888 31887217

[R23] Charon J. et al. (2022) ‘RdRp-scan: A Bioinformatic Resource to Identify and Annotate Divergent RNA Viruses in Metagenomic Sequence Data’, *Virus Evolution*, 8: veac082. 10.1093/ve/veac082 PMC9752661 36533143

[R24] Charon J., Murray S., and Holmes E. C. (2021) ‘Revealing RNA Virus Diversity and Evolution in Unicellular Algae Transcriptomes’, *Virus Evolution*, 7(2): veab070. 10.1093/ve/veab070 PMC9927876 36819971

[R25] Chau S. et al. (2023) ‘Diverse Yeast Antiviral Systems Prevent Lethal Pathogenesis Caused by the L-A Mycovirus’, *Proceedings of the National Academy of Sciences of the United States of America*, 120: e2208695120. 10.1073/pnas.2208695120 PMC10089162 36888656

[R26] Chen L. et al. (2021) ‘Transcriptomics Analysis Reveals the Immune Response Mechanism of Rabbits with Diarrhea Fed an Antibiotic-Free Diet’, *Animals (Basel)*, 11: 2994. 10.3390/ani11102994 PMC8532911 34680013

[R27] Chen Y.-M. et al. (2022) ‘RNA Viromes from Terrestrial Sites across China Expand Environmental Viral Diversity’, *Nature Microbiology*, 7: 1312–23. 10.1038/s41564-022-01180-2 35902778

[R28] Chen -X.-X., Wei-Chen W., and Shi M. (2021) ‘Discovery and Characterization of Actively Replicating DNA and Retro-Transcribing Viruses in Lower Vertebrate Hosts Based on RNA Sequencing’, *Viruses*, 13: 1042. 10.3390/v13061042 PMC8227577 34072878

[R29] Chiba Y. et al. (2021) ‘Discovery of Divided RdRp Sequences and a Hitherto Unknown Genomic Complexity in Fungal Viruses’, *Virus Evolution*, 7(1): veaa101. 10.1093/ve/veaa101 PMC7816673 33505709

[R30] Chiba Y. et al. (2023) ‘The First Identification of a Narnavirus in Bigyra, a Marine Protist’, *Microbes & Environments*, 38: ME22077. 10.1264/jsme2.ME22077 PMC10037099 36858534

[R31] Crooks G. E. et al. (2004) ‘WebLogo: A Sequence Logo Generator’, *Genome Research*, 14: 1188–90. 15173120 10.1101/gr.849004 PMC419797

[R32] Cui R. et al. (2022) ‘Integrated Analysis of the Whole Transcriptome of Skeletal Muscle Reveals the ceRNA Regulatory Network Related to the Formation of Muscle Fibers in Tan Sheep’, *Frontiers in Genetics*, 13: 991606. 10.3389/fgene.2022.991606 PMC9624228 36330447

[R33] Dardé M.-L. (2020) ‘Chapter 3 - Molecular epidemiology and population structure of Toxoplasma gondii,’ in Louis, M. W. and Kim, K. (eds.) *Toxoplasma gondii*, 3rd edn. pp. 63–116. London: Academic Press.

[R34] Dardé M. L. et al. (1998) ‘Severe Toxoplasmosis Caused by a Toxoplasma Gondii Strain with a New Isoenzyme Type Acquired in French Guyana’, *Journal of Clinical Microbiology*, 36: 324. 10.1128/jcm.36.1.324-324.1998 PMC124868 9431981

[R35] de Carvalho R. V. H. et al. (2019) ‘Leishmania RNA Virus Exacerbates Leishmaniasis by Subverting Innate Immunity via TLR3-mediated NLRP3 Inflammasome Inhibition’, *Nature Communications*, 10: 5273. 10.1038/s41467-019-13356-2 PMC6872735 31754185

[R36] Deng S. et al. (2023) ‘Cryptosporidium Uses CSpV1 to Activate Host Type I Interferon and Attenuate Antiparasitic Defenses’, *Nature Communications*, 14: 1456. 10.1038/s41467-023-37129-0 PMC10020566 36928642

[R37] Dubey J. P. (2021) ‘Outbreaks of Clinical Toxoplasmosis in Humans: Five Decades of Personal Experience, Perspectives and Lessons Learned’, *Parasites and Vectors*, 14: 263. 10.1186/s13071-021-04769-4 PMC8136135 34011387

[R136] ——— (2021) *Toxoplasmosis of Animals and Humans*, 3rd. London, England: CRC Press.

[R38] Dubey J. P. et al. (2008) ‘Isolation and Genetic Characterization of Toxoplasma Gondii from Raccoons (Procyon Lotor), Cats (Felis Domesticus), Striped Skunk (Mephitis Mephitis), Black Bear (Ursus Americanus), and Cougar (Puma Concolor) from Canada’, *Journal of Parasitology*, 94: 42–5. 18372620 10.1645/GE-1349.1

[R39] Edgar R. C. (2004) ‘MUSCLE: Multiple Sequence Alignment with High Accuracy and High Throughput’, *Nucleic Acids Research*, 32: 1792–7. 15034147 10.1093/nar/gkh340 PMC390337

[R40] Edgar R. C. et al. (2022) ‘Petabase-Scale Sequence Alignment Catalyses Viral Discovery’, *Nature*, 602: 142–7. 35082445 10.1038/s41586-021-04332-2

[R41] Eren R. O. et al. (2016) ‘Mammalian Innate Immune Response to a Leishmania-Resident RNA Virus Increases Macrophage Survival to Promote Parasite Persistence’, *Cell Host & Microbe*, 20: 318–28. 27593513 10.1016/j.chom.2016.08.001 PMC5493041

[R42] Espino-Vázquez A. N. et al. (2020) ‘Narnaviruses: Novel Players in Fungal-Bacterial Symbioses’, *The ISME Journal*, 14: 1743–54. 32269378 10.1038/s41396-020-0638-y PMC7305303

[R43] Fichorova R. N. et al. (2012) ‘Endobiont Viruses Sensed by the Human Host - Beyond Conventional Antiparasitic Therapy’, *PLoS One*, 7: e48418. 10.1371/journal.pone.0048418 PMC3492353 23144878

[R44] Finn R. D. et al. (2014) ‘Pfam: The Protein Families Database’, *Nucleic Acids Research*, 42: D222–30. 24288371 10.1093/nar/gkt1223 PMC3965110

[R45] Fitzgerald K. A., and Kagan J. C. (2020) ‘Toll-Like Receptors and the Control of Immunity’, *Cell*, 180: 1044–66. 32164908 10.1016/j.cell.2020.02.041 PMC9358771

[R46] Forgia M. et al. (2023) ‘Hybrids of RNA Viruses and Viroid-Like Elements Replicate in Fungi’, *Nature Communications*, 14: 2591. 10.1038/s41467-023-38301-2 PMC10162972 37147358

[R47] Gajria B. et al. (2008) ‘ToxoDB: An Integrated Toxoplasma Gondii Database Resource’, *Nucleic Acids Research*, 36: D553–6. 18003657 10.1093/nar/gkm981 PMC2238934

[R48] Gao Y. et al. (2021) ‘Full-Length Transcriptome Sequence Analysis of Eimeria Necatrix Unsporulated Oocysts and Sporozoites Identifies Genes Involved in Cellular Invasion’, *Veterinary Parasitology*, 296: 109480. 10.1016/j.vetpar.2021.109480 34120030

[R49] Garvik B., and Haber J. E. (1978) ‘New Cytoplasmic Genetic Element that Controls 20S RNA Synthesis during Sporulation in Yeast’, *Journal of Bacteriology*, 134: 261–9. 348682 10.1128/jb.134.1.261-269.1978 PMC222242

[R50] Gil J. C., and Hird S. M. (2022) ‘Multiomics Characterization of the Canada Goose Fecal Microbiome Reveals Selective Efficacy of Simulated Metagenomes’, *Microbiology Spectrum*, 10: e0238422. 10.1128/spectrum.02384-22 PMC9769641 36318011

[R51] Goh E. J. H. et al. (2023) ‘Ocular Toxoplasmosis’, *Ocular Immunology and Inflammation*, 31: 1342–61. 36095008 10.1080/09273948.2022.2117705

[R52] Gómez-Arreaza A. et al. (2017) ‘Viruses of Parasites as Actors in the Parasite-Host Relationship: A ‘Ménage À Trois’’, *Acta Tropica*, 166: 126–32. 27876650 10.1016/j.actatropica.2016.11.028

[R53] Gong P., and Peersen O. B. (2010) ‘Poliovirus Polymerase Elongation Complex’, Worldwide Protein Data Bank.

[R54] Grybchuk D. et al. (2018) ‘Viral Discovery and Diversity in Trypanosomatid Protozoa with a Focus on Relatives of the Human Parasite *Leishmania*’, *Proceedings of the National Academy of Sciences of the United States of America*, 115: E506–15. 29284754 10.1073/pnas.1717806115 PMC5776999

[R55] Harvey E. et al. (2019) ‘Identification of Diverse Arthropod Associated Viruses in Native Australian Fleas’, *Virology*, 535: 189–99. 31319276 10.1016/j.virol.2019.07.010

[R56] Hassan M. A. et al. (2019) ‘Clonal and Atypical Toxoplasma Strain Differences in Virulence Vary with Mouse Sub-Species’, *International Journal for Parasitology*, 49: 63–70. 30471286 10.1016/j.ijpara.2018.08.007 PMC6344230

[R57] He K. et al. (2021) ‘Echolocation in Soft-Furred Tree Mice’, *Science*, 372: eaay1513. 10.1126/science.aay1513 34140356

[R58] Heeren S. et al. (2023) ‘Diversity and Dissemination of Viruses in Pathogenic Protozoa’, *Nature Communications*, 14: 8343. 10.1038/s41467-023-44085-2 PMC10724245 38102141

[R59] Heneka M. T. et al. (2015) ‘Neuroinflammation in Alzheimer’s Disease’, *The Lancet Neurology*, 14: 388–405. 25792098 10.1016/S1474-4422(15)70016-5 PMC5909703

[R60] Hillman B. I., and Cai G. (2013) ‘The Family Narnaviridae,’ in Ghabrial, S. A.(ed.) *Advances in Virus Research*, pp. 149–76. Radarweg, Amsterdam, Netherlands: Elsevier. 10.1016/B978-0-12-394315-6.00006-4 23498906

[R61] Hillman B. I. Esteban R. (2011) ‘Family - Narnaviridae’, in King, A. M. Q. et al. (eds) *Virus Taxonomy*, Vol. 9, pp. 1055–60. Netherlands: Elsevier.

[R62] Hoang D. T. et al. (2018) ‘UFBoot2: Improving the Ultrafast Bootstrap Approximation’, *Molecular Biology and Evolution*, 35: 518–22. 29077904 10.1093/molbev/msx281 PMC5850222

[R63] Hou X. (2023) ‘Using Artificial Intelligence to Document the Hidden RNA Virosphere,’ *bioRxiv*. 10.1016/j.cell.2024.09.027 39389057

[R64] Hu Z. et al. (2022) ‘Inflammasome Activation Dampens Type I IFN Signaling to Strengthen Anti-Toxoplasma Immunity’, *MBio*, 13: e02361–22. 36214572 10.1128/mbio.02361-22 PMC9765454

[R65] Ives A. et al. (2011) ‘Leishmania RNA Virus Controls the Severity of Mucocutaneous Leishmaniasis’, *Science*, 331: 775–8. 21311023 10.1126/science.1199326 PMC3253482

[R66] Jensen S., and Randrup Thomsen A. (2012) ‘Sensing of RNA Viruses: A Review of Innate Immune Receptors Involved in Recognizing RNA Virus Invasion’, *Journal of Virology*, 86: 2900–10. 22258243 10.1128/JVI.05738-11 PMC3302314

[R67] Jones K. E. et al. (2008) ‘Global Trends in Emerging Infectious Diseases’, *Nature*, 451: 990–3. 18288193 10.1038/nature06536 PMC5960580

[R68] Jones P. et al. (2014) ‘InterProScan 5: Genome-Scale Protein Function Classification’, *Bioinformatics*, 30: 1236–40. 24451626 10.1093/bioinformatics/btu031 PMC3998142

[R69] Jung B.-K. et al. (2012) ‘Toxoplasma Gondii Infection in the Brain Inhibits Neuronal Degeneration and Learning and Memory Impairments in a Murine Model of Alzheimer’s Disease’, *PLoS One*, 7: e33312. 10.1371/journal.pone.0033312 PMC3310043 22470449

[R70] Jurrus E. et al. (2018) ‘Improvements to the APBS Biomolecular Solvation Software Suite’, *Protein Science*, 27: 112–28. 28836357 10.1002/pro.3280 PMC5734301

[R71] Kadowaki K., and Halvorson H. O. (1971) ‘Appearance of a New Species of Ribonucleic Acid during Sporulation in Saccharomyces Cerevisiae’, *Journal of Bacteriology*, 105: 826–30. 5547990 10.1128/jb.105.3.826-830.1971 PMC248506

[R72] Kalyaanamoorthy S. et al. (2017) ‘ModelFinder: Fast Model Selection for Accurate Phylogenetic Estimates’, *Nature Methods*, 14: 587–9. 28481363 10.1038/nmeth.4285 PMC5453245

[R73] Kassambara A. (2023) ‘Ggpubr: ‘Ggplot2’ Based Publication Ready Plots’, <https://rpkgs.datanovia.com/ggpubr/> accessed 18 Sept 2023.

[R74] Katz K. S. et al. (2021) ‘STAT: A Fast, Scalable, MinHash-based k-Mer Tool to Assess Sequence Read Archive Next-Generation Sequence Submissions’, *Genome Biology*, 22: 270. 10.1186/s13059-021-02490-0 PMC8450716 34544477

[R75] Keller L. M., and Weber-Ban E. (2023) ‘An Emerging Class of Nucleic Acid-Sensing Regulators in Bacteria: WYL Domain-Containing Proteins’, *Current Opinion in Microbiology*, 74: 102296. 10.1016/j.mib.2023.102296 37027901

[R76] Kempen M. V. et al. (2023) ‘Fast and Accurate Protein Structure Search with Foldseek’, *Nature Biotechnology*, 42: 243–6. 10.1038/s41587-023-01773-0 PMC10869269 37156916

[R77] Khan A. et al. (2009) ‘Selection at a Single Locus Leads to Widespread Expansion of Toxoplasma Gondii Lineages that are Virulent in Mice’, *PLoS Genetics*, 5: e1000404. 10.1371/journal.pgen.1000404 PMC2644818 19266027

[R78] Khan Mirzaei M. et al. (2021) ‘Challenges of Studying the Human Virome - Relevant Emerging Technologies’, *Trends in Microbiology*, 29: 171–81. 32622559 10.1016/j.tim.2020.05.021

[R79] Khurana S., and Batra N. (2016) ‘Toxoplasmosis in Organ Transplant Recipients: Evaluation, Implication, and Prevention’, *Tropical Parasitology*, 6: 123–8. 27722100 10.4103/2229-5070.190814 PMC5048698

[R80] Kim D. et al. (2019) ‘Graph-Based Genome Alignment and Genotyping with HISAT2 and HISAT-genotype’, *Nature Biotechnology*, 37: 907–15. 10.1038/s41587-019-0201-4 PMC7605509 31375807

[R81] Koonin E. V. et al. (2020) ‘Global Organization and Proposed Megataxonomy of the Virus World’, *Microbiology and Molecular Biology Reviews*, 84: 10.1128/mmbr.00061-19. 10.1128/MMBR.00061-19 PMC7062200 32132243

[R82] Kuhlmann F. M. et al. (2017) ‘Antiviral Screening Identifies Adenosine Analogs Targeting the Endogenous dsRNA *Leishmania* RNA Virus 1 (LRV1) Pathogenicity Factor’, *Proceedings of the National Academy of Sciences of the United States of America*, 114: E811–19. 28096399 10.1073/pnas.1619114114 PMC5293060

[R83] Kumar R. et al. (2020) ‘Type I Interferons Suppress Anti-Parasitic Immunity and Can Be Targeted to Improve Treatment of Visceral Leishmaniasis’, *Cell Reports*, 30: 2512–2525.e9. 32101732 10.1016/j.celrep.2020.01.099 PMC7981274

[R84] Kuroki M. et al. (2023) ‘Experimental Verification of Strain-Dependent Relationship between Mycovirus and Its Fungal Host’, *iScience*, 26: 107337. 10.1016/j.isci.2023.107337 PMC10372822 37520716

[R85] Langmead B., and Salzberg S. L. (2012) ‘Fast Gapped-Read Alignment with Bowtie 2’, *Nature Methods*, 9: 357–9. 22388286 10.1038/nmeth.1923 PMC3322381

[R86] Lanz T. V. et al. (2022) ‘Clonally Expanded B Cells in Multiple Sclerosis Bind EBV EBNA1 and GlialCAM’, *Nature*, 603: 321–7. 35073561 10.1038/s41586-022-04432-7 PMC9382663

[R87] Laskowski R. A. et al. (2018) ‘PDBsum: Structural Summaries of PDB Entries’, *Protein Science*, 27: 129–34. 28875543 10.1002/pro.3289 PMC5734310

[R88] Lee B. D. et al. (2023) ‘Mining Metatranscriptomes Reveals a Vast World of Viroid-Like Circular RNAs’, *Cell*, 186: 646–661.e4. 36696902 10.1016/j.cell.2022.12.039 PMC9911046

[R89] Lee A. J., and Ashkar A. A. (2018) ‘The Dual Nature of Type I and Type II Interferons’, *Frontiers in Immunology*, 9: 2061. 10.3389/fimmu.2018.02061 PMC6141705 30254639

[R90] Leng F., and Edison P. (2021) ‘Neuroinflammation and Microglial Activation in Alzheimer Disease: Where Do We Go from Here?’, *Nature Reviews Neurology*, 17: 157–72. 33318676 10.1038/s41582-020-00435-y

[R91] Li Y. et al. (2019) ‘Persistent Toxoplasma Infection of the Brain Induced Neurodegeneration Associated with Activation of Complement and Microglia’, *Infection and Immunity*, 87: 10–128. 10.1128/IAI.00139-19 PMC6652752 31182619

[R92] Liang G., and Bushman F. D. (2021) ‘The Human Virome: Assembly, Composition and Host Interactions’, *Nature Reviews, Microbiology*, 19: 514–27. 33785903 10.1038/s41579-021-00536-5 PMC8008777

[R93] Liao Y., Smyth G. K., and Shi W. (2014) ‘featureCounts: An Efficient General Purpose Program for Assigning Sequence Reads to Genomic Features’, *Bioinformatics*, 30: 923–30. 24227677 10.1093/bioinformatics/btt656

[R94] Liberzon A. et al. (2015) ‘The Molecular Signatures Database (Msigdb) Hallmark Gene Set Collection’, *Cell Systems*, 1: 417–25. 26771021 10.1016/j.cels.2015.12.004 PMC4707969

[R95] Li H., and Durbin R. (2010) ‘Fast and Accurate Long-Read Alignment with Burrows-Wheeler Transform’, *Bioinformatics*, 26: 589–95. 20080505 10.1093/bioinformatics/btp698 PMC2828108

[R96] Lorenzi H. et al. (2016) ‘Local Admixture of Amplified and Diversified Secreted Pathogenesis Determinants Shapes Mosaic Toxoplasma Gondii Genomes’, *Nature Communications*, 7: 10147. 10.1038/ncomms10147 PMC4729833 26738725

[R97] Love M. I., Huber W., and Anders S. (2014) ‘Moderated Estimation of Fold Change and Dispersion for RNA-seq Data with DESeq2’, *Genome Biology*, 15: 550. 10.1186/s13059-014-0550-8 PMC4302049 25516281

[R98] Lye L.-F. et al. (2016) ‘A Narnavirus -like Element from the Trypanosomatid Protozoan Parasite Leptomonas Seymouri’, *Genome Announcements*, 4: 10-128. 10.1128/genomeA.00713-16 PMC4974305 27491984

[R99] Matta S. K. et al. (2019) ‘Toxoplasma Gondii Effector TgIST Blocks Type I Interferon Signaling to Promote Infection’, *Proceedings of the National Academy of Sciences of the United States of America*, 116: 17480–91. 31413201 10.1073/pnas.1904637116 PMC6717281

[R100] McGovern K. E. et al. (2020) ‘Aging with Toxoplasma Gondii Results in Pathogen Clearance, Resolution of Inflammation, and Minimal Consequences to Learning and Memory’, *Scientific Reports*, 10: 7979. 10.1038/s41598-020-64823-6 PMC7224383 32409672

[R101] Melo M. B. et al. (2013) ‘Transcriptional Analysis of Murine Macrophages Infected with Different Toxoplasma Strains Identifies Novel Regulation of Host Signaling Pathways’, *PLoS Pathogens*, 9: e1003779. 10.1371/journal.ppat.1003779 PMC3868521 24367253

[R102] Miller M. A. et al. (2023) ‘Newly Detected, Virulent Toxoplasma Gondii COUG Strain Causing Fatal Steatitis and Toxoplasmosis in Southern Sea Otters (Enhydra Lutris Nereis)’, *Frontiers in Marine Science*, 10: 1116899.

[R103] Miller R. L., Wang A. L., and Wang C. C. (1988) ‘Identification of Giardia Lamblia Isolates Susceptible and Resistant to Infection by the Double-Stranded RNA Virus’, *Experimental Parasitology*, 66: 118–23. 3366209 10.1016/0014-4894(88)90056-2

[R104] Minh B. Q. et al. (2020) ‘IQ-TREE 2: New Models and Efficient Methods for Phylogenetic Inference in the Genomic Era’, *Molecular Biology and Evolution*, 37: 1530–4. 32011700 10.1093/molbev/msaa015 PMC7182206

[R105] Mirdita M. et al. (2022) ‘ColabFold: Making Protein Folding Accessible to All’, *Nature Methods*, 19: 679–82. 35637307 10.1038/s41592-022-01488-1 PMC9184281

[R106] Narayanasamy R. K. et al. (2022) ‘Cytidine Nucleoside Analog Is an Effective Antiviral Drug against Trichomonasvirus’, *Journal of Microbiology, Immunology and Infection*, 55: 191–8. 10.1016/j.jmii.2021.08.008 34479802

[R107] Nayeri Chegeni T. et al. (2019) ‘Is Toxoplasma Gondii A Potential Risk Factor for Alzheimer’s Disease? A Systematic Review and Meta-Analysis’, *Microbial Pathogenesis*, 137: 103751. 10.1016/j.micpath.2019.103751 31536800

[R108] Neri U. et al. (2022) ‘Expansion of the Global RNA Virome Reveals Diverse Clades of Bacteriophages’, *Cell*, 185: 4023–4037.e18. 36174579 10.1016/j.cell.2022.08.023

[R109] Ngô H. M. et al. (2017) ‘Toxoplasma Modulates Signature Pathways of Human Epilepsy, Neurodegeneration & Cancer’, *Scientific Reports*, 7: 11496. 10.1038/s41598-017-10675-6 PMC5597608 28904337

[R110] Niedelman W. et al. (2012) ‘The Rhoptry Proteins ROP18 and ROP5 Mediate Toxoplasma Gondii Evasion of the Murine, but Not the Human, Interferon-Gamma Response’, *PLoS Pathogens*, 8: e1002784. 10.1371/journal.ppat.1002784 PMC3386190 22761577

[R111] Nimgaonkar V. L. et al. (2016) ‘Temporal Cognitive Decline Associated with Exposure to Infectious Agents in a Population-Based, Aging Cohort’, *Alzheimer Disease & Associated Disorders*, 30: 216–22. 26710257 10.1097/WAD.0000000000000133 PMC4919236

[R112] Olendraite I., Brown K., and Firth A. E. (2023) ‘Identification of RNA Virus-Derived RdRp Sequences in Publicly Available Transcriptomic Data Sets’, *Molecular Biology and Evolution*, 40: msad060. 10.1093/molbev/msad060 PMC10101049 37014783

[R113] Ortiz-Guerrero G. et al. (2020) ‘Pathophysiological Mechanisms of Cognitive Impairment and Neurodegeneration by Toxoplasma Gondii Infection’, *Brain Sciences*, 10: 369. 10.3390/brainsci10060369 PMC7349234 32545619

[R114] Pappas G., Roussos N., and Falagas M. E. (2009) ‘Toxoplasmosis Snapshots: Global Status of Toxoplasma Gondii Seroprevalence and Implications for Pregnancy and Congenital Toxoplasmosis’, *International Journal for Parasitology*, 39: 1385–94. 19433092 10.1016/j.ijpara.2009.04.003

[R115] Pu X. et al. (2021) ‘Giardia Duodenalis Induces Proinflammatory Cytokine Production in Mouse Macrophages via TLR9-mediated P38 and ERK Signaling Pathways’, *Frontiers in Cell and Developmental Biology*., 9: 694675. 10.3389/fcell.2021.694675 PMC8319647 34336841

[R116] Rehwinkel J., and Gack M. U. (2020) ‘RIG-I-like Receptors: Their Regulation and Roles in RNA Sensing’, *Nature Reviews Immunology*, 20: 537–51. 10.1038/s41577-020-0288-3 PMC7094958 32203325

[R117] Rhie A. et al. (2021) ‘Towards Complete and Error-Free Genome Assemblies of All Vertebrate Species’, *Nature*, 592: 737–46. 33911273 10.1038/s41586-021-03451-0 PMC8081667

[R118] Robinson J. T. et al. (2011) ‘Integrative Genomics Viewer’, *Nature Biotechnology*, 29: 24–6. 10.1038/nbt.1754 PMC3346182 21221095

[R119] Rodrigues J. R., Roy S. W., and Sehgal R. N. M. (2022) ‘Novel RNA Viruses Associated with Avian Haemosporidian Parasites’, *PLoS One*, 17: e0269881. 10.1371/journal.pone.0269881 PMC9246168 35771829

[R120] Roy E. R. et al. (2020) ‘Type I Interferon Response Drives Neuroinflammation and Synapse Loss in Alzheimer Disease’, *Journal of Clinical Investigation*, 130: 1912–30. 31917687 10.1172/JCI133737 PMC7108898

[R121] Roy E. R. et al. (2022) ‘Concerted Type I Interferon Signaling in Microglia and Neural Cells Promotes Memory Impairment Associated with AmyloidPlaques’, *Immunity*, 55: 879–894.e6. 35443157 10.1016/j.immuni.2022.03.018 PMC9109419

[R122] Sanford S. A. I. et al. (2023) ‘The Type-i Interferon Response Potentiates Seeded Tau Aggregation and Exacerbates Tau Pathology’, *Alzheimers Dement*, 20: 1013–25. 37849026 10.1002/alz.13493 PMC10916982

[R123] Schoenemeyer A. et al. (2005) ‘The Interferon Regulatory Factor, IRF5, Is a Central Mediator of Toll-Like Receptor 7 Signaling’, *Journal of Biological Chemistry*, 280: 17005–12. 15695821 10.1074/jbc.M412584200

[R124] Schrödinger L. L. C. (2015) The PyMOL Molecular Graphics System, Version 2.0. <https://pymol.org/support.html>.

[R125] Shen W. et al. (2016) ‘SeqKit: A Cross-Platform and Ultrafast Toolkit for FASTA/Q File Manipulation’, *PLoS One*, 11: e0163962. 10.1371/journal.pone.0163962 PMC5051824 27706213

[R126] Shi M. et al. (2016) ‘Redefining the Invertebrate RNA Virosphere’, *Nature*, 540: 539–43. 27880757 10.1038/nature20167

[R127] Shi M. et al. (2018) ‘The Evolutionary History of Vertebrate RNA Viruses’, *Nature*, 556: 197–202. 29618816 10.1038/s41586-018-0012-7

[R128] Silva-Barrios S., and Stäger S. (2017) ‘Protozoan Parasites and Type I IFNs’, *Frontiers in Immunology*, 8: 14. 10.3389/fimmu.2017.00014 PMC5243830 28154565

[R129] Soldan S. S., and Lieberman P. M. (2023) ‘Epstein-Barr Virus and Multiple Sclerosis’, *Nature Reviews, Microbiology*, 21: 51–64. 35931816 10.1038/s41579-022-00770-5 PMC9362539

[R130] Su C. et al. (2003) ‘Recent Expansion of Toxoplasma through Enhanced Oral Transmission’, *Science*, 299: 414–6. 12532022 10.1126/science.1078035

[R131] Subramanian A. et al. (2005) ‘Gene Set Enrichment Analysis: A Knowledge-Based Approach for Interpreting Genome-Wide Expression Profiles’, *Proceedings of the National Academy of Sciences of the United States of America*, 102: 15545–50. 16199517 10.1073/pnas.0506580102 PMC1239896

[R132] Sukla S. et al. (2017) ‘Leptomonas Seymouri Narna-Like Virus 1 and Not Leishmaniaviruses Detected in Kala-Azar Samples from India’, *Archives of Virology*, 162: 3827–35. 28939968 10.1007/s00705-017-3559-y

[R133] Sun Y. et al. (2022) ‘Genome-Wide Characterization of lncRNAs and mRNAs in Muscles with Differential Intramuscular Fat Contents’, *Frontiers in Veterinary Science*, 9: 982258. 10.3389/fvets.2022.982258 PMC9393339 36003408

[R134] Tian L. et al. (2021) ‘RNA-dependent RNA Polymerase (Rdrp) Inhibitors: The Current Landscape and Repurposing for the COVID-19 Pandemic’, *European Journal of Medicinal Chemistry*, 213: 113201. 10.1016/j.ejmech.2021.113201 PMC7826122 33524687

[R135] Torniainen-Holm M. et al. (2019) ‘The Lack of Association between Herpes Simplex Virus 1 or Toxoplasma Gondii Infection and Cognitive Decline in the General Population: An 11-Year Follow-up Study’, *Brain, Behavior, and Immunity*, 76: 159–64. 30465879 10.1016/j.bbi.2018.11.016

[R137] Tyebji S. et al. (2019) ‘Toxoplasmosis: A Pathway to Neuropsychiatric Disorders’, *Neuroscience and Biobehavioral Reviews*, 96: 72–92. 30476506 10.1016/j.neubiorev.2018.11.012

[R138] Venkataraman S., Prasad B., and Selvarajan R. (2018) ‘RNA Dependent RNA Polymerases: Insights from Structure, Function and Evolution’, *Viruses*, 10: 76. 10.3390/v10020076 PMC5850383 29439438

[R139] Vijayraghavan S. et al. (2023) ‘A Novel Narnavirus Is Widespread in Saccharomyces Cerevisiae and Impacts Multiple Host Phenotypes’, *G3: Genes, Genomes, Genetics*, 13(2): GALE|A777680148. 10.1093/g3journal/jkac337 PMC9911063 36560866

[R140] Wejksnora P. J., and Haber J. E. (1978) ‘Ribonucleoprotein Particle Appearing during Sporulation in Yeast’, *Journal of Bacteriology*, 134: 246–60. 348681 10.1128/jb.134.1.246-260.1978 PMC222241

[R141] Wen Y. et al. (2022) ‘Comparative Transcriptome Analysis Reveals the Mechanism Associated with Dynamic Changes in Meat Quality of the Longissimus Thoracis Muscle in Tibetan Sheep at Different Growth Stages’, *Frontiers in Veterinary Science*, 9: 926725. 10.3389/fvets.2022.926725 PMC9298548 35873690

[R142] Wickham H. (2016) *Ggplot2: Elegant Graphics for Data Analysis*. New York: Springer.

[R143] Wilkins D. (2020) ‘Gggenes: Draw Gene Arrow Maps in ‘Ggplot2’’, <https://CRAN.R-project.org/package=gggenes> accessed 18 Sept 2023.

[R144] Wolf Y. I. et al. (2020) ‘Doubling of the Known Set of RNA Viruses by Metagenomic Analysis of an Aquatic Virome’, *Nature Microbiology*, 5: 1262–70. 10.1038/s41564-020-0755-4 PMC7508674 32690954

[R145] Woolhouse M. E. J., and Adair K. (2013) ‘The Diversity of Human RNA Viruses’, *Future Virology*, 8: 159–71. 29503665 10.2217/fvl.12.129 PMC5831953

[R146] Wu Z. et al. (2021) ‘Decoding the RNA Viromes in Rodent Lungs Provides New Insight into the Origin and Evolutionary Patterns of Rodent-Borne Pathogens in Mainland Southeast Asia’, *Microbiome*, 9: 18. 10.1186/s40168-020-00965-z PMC7818139 33478588

[R147] Wyman C. P. et al. (2017) ‘Association between Toxoplasma Gondii Seropositivity and Memory Function in Nondemented Older Adults’, *Neurobiology of Aging*, 53: 76–82. 28235681 10.1016/j.neurobiolaging.2017.01.018 PMC5482532

[R148] Xiao J., Savonenko A., and Yolken R. H. (2022) ‘Strain-Specific Pre-Existing Immunity: A Key to Understanding the Role of Chronic Toxoplasma Infection in Cognition and Alzheimer’s Diseases?’, *Neuroscience and Biobehavioral Reviews*, 137: 104660. 10.1016/j.neubiorev.2022.104660 35405182

[R149] Xie Y. et al. (2021) ‘Global Transcriptome Landscape of the Rabbit Protozoan Parasite Eimeria Stiedae’, *Parasites and Vectors*, 14: 308. 10.1186/s13071-021-04811-5 PMC8186055 34099031

[R150] Xu Z. et al. (2022) ‘Virome of Bat-Infesting Arthropods: Highly Divergent Viruses in Different Vectors’, *Journal of Virology*, 96: e0146421. 10.1128/jvi.01464-21 PMC8865543 34586860

[R151] Yang H.-Y. et al. (2021) ‘Risk of Dementia in Patients with Toxoplasmosis: A Nationwide, Population-Based Cohort Study in Taiwan’, *Parasites and Vectors*, 14: 435. 10.1186/s13071-021-04928-7 PMC8401101 34454590

[R152] Yang X. et al. (2023) ‘Meta-Viromic Sequencing Reveals Virome Characteristics of Mosquitoes and Culicoides on Zhoushan Island, China’, *Microbiology Spectrum*, 11: e0268822. 10.1128/spectrum.02688-22 PMC9927462 36651764

[R153] Yu G. et al. (2016) ‘Ggtree: An R Package for Visualization and Annotation of Phylogenetic Trees with Their Covariates and Other Associated Data’, *Methods in Ecology and Evolution*, 8.

[R154] Zayed A. A. et al. (2022) ‘Cryptic and Abundant Marine Viruses at the Evolutionary Origins of Earth’s RNA Virome’, *Science*, 376: 156–62. 35389782 10.1126/science.abm5847 PMC10990476

[R155] Zhang Y., and Skolnick J. (2005) ‘TM-align: A Protein Structure Alignment Algorithm Based on the TM-score’, *Nucleic Acids Research*, 33: 2302–9. 15849316 10.1093/nar/gki524 PMC1084323

[R156] Zhao Z. et al. (2023) ‘Multiple Regulations of Parasitic Protozoan Viruses: A Double-Edged Sword for Protozoa’, *MBio*, 14: e0264222. 10.1128/mbio.02642-22 PMC9973342 36633419

[R157] Zheludev I. N. et al. (2024) ‘Viroid-Like Colonists of Human Microbiomes’, *bioRxiv*. 10.1016/j.cell.2024.09.033 PMC11949080 39481381

[R158] Zhou H. et al. (2021) ‘Identification of Novel Bat Coronaviruses Sheds Light on the Evolutionary Origins of SARS-CoV-2 and Related Viruses’, *Cell*, 184: 4380–4391.e14. 34147139 10.1016/j.cell.2021.06.008 PMC8188299

[R159] Zhou L. et al. (2022a) ‘Ggmsa: A Visual Exploration Tool for Multiple Sequence Alignment and Associated Data’, *Briefings in Bioinformatics*, 23: bbac222. 10.1093/bib/bbac222 35671504

[R160] Zhou Z. et al. (2022b) ‘Expression Profile Analysis to Identify Circular RNA Expression Signatures in Muscle Development of Wu’an Goat Longissimus Dorsi Tissues’, *Frontiers in Veterinary Science*, 9: 833946. 10.3389/fvets.2022.833946 PMC9062782 35518637

