# Supplementary material for: A parasite odyssey: An RNA virus concealed in Toxoplasma gondii
Source: Virus Evol. 2024 May 11;10(1):veae040. doi: 10.1093/ve/veae040 (PMC11137675; doi:10.1093/ve/veae040)
Supplement: veae040_Supp [file veae040_supp.zip › suppl_data/ao_supplement_legend.docx]

Supplementary Text: A Parasite Odyssey:
An RNA virus concealed in *Toxoplasma gondii*

Purav Gupta^1,2,3*^ , Aiden Hiller^2,3,*^, Jawad Chowdhury^2,3,*^, Declan Lim^2,3,*^,

Dillon Yee Lim^4,*^, Jeroen P.J. Saeij^5,*^, Artem Babaian^2,3,*†^, Felipe Rodriguez^5,*^,

Luke Pereira^2,3,*^, Alex Morales^2,3,*^

# Supplementary Figure Legends

**Supplementary Figure S1: Maximum likelihood tree for T. gondii transcriptomes.**

RNAseq reads were aligned to the T. gondii ME49 reference genome (version 64) from each of the 31 T. gondii samples of the Melo study (Melo et al., 2013). Phylogeny was constructed from consensus single nucleotide polymorphisms (SNPs) in expressed annotated exons. 8,201,735/30,124,248 (27.22% ) exonic sites were expressed across all samples of which a further 156,773/8,201,735 (1.91%) sites were polymorphic in at least one sample. IQ-TREE (v2.2.2.6; iqtree2 -s melo.snp-only nogap msa.fa -m TEST+ASC -bb 1000 -alrt 1000; model: TVM+F+ASC+G4) was ran. Nodes supported by 100% bootstraps are indicated with a black circle

**Supplementary Figure S2: Melo study T. gondii transcriptomes sample contamination.**

RNAseq reads were aligned to the T. gondii ME49 reference genome (version 64) from each of the 31 T. gondii samples of the Melo study (Melo et al., 2013). To test if the presence of Ao in RUB and COUGAR was the result of sample cross-contamination, we manually inspected reads from the Melo transcriptomes in IGV 2.16.0. The ME49 strain is included as an Ao-negative control related to COUGAR (Lorenzi clade D). RUB-specific alleles were not present above the sequencing-error background (1 %) in the COUGER sample or vice versa. Where possible, the reads for potential cross-contaminating alleles were inspected at adjacent linked sites. Manual inspection of several dozen sites revealed no read-level evidence for cross-contamination of samples.

**Supplementary Figure S3: Pairwise percent amino acid identity between narnavirus RdRp.**

**A.** Heatmap plotting the pairwise percent identity for each RdRp in the narnavirus phylogeny. Percent identity was calculated on the basis of pairwise alignments for the RdRp core (motifs F-E) and thumb, gaps were not penalized. The heatmap is symmetrical (i.e. RdRps on the diagonal are identical), with tentative phylogenetic groupings shown. **B.** Scatterplot of between-Apocryptovirus (pairwise) percent identity for RDRP, pORF1 or pORF2 where contig 1 and contig 2 association was unambiguous. USEARCH v11 was run with parameters ‘-allpairs global -acceptall’. Scores were extracted using Pandas and plotted with Matplotlib v3.8 (Hunter, 2007). **C.** Pearson correlation of GC content across contigs 1 and 2 for each Apocryptovirus species. A. lentulus Narnavirus 1 is also included as a closely-related bi-segmented narnavirus.

**Supplementary Figure S4: Narnavirus RdRp phylogeny with Apocrytoviruses.**

Maximum-likelihood phylogenetic tree of A. odysseus and the related Apocryptoviruses estimated based on RdRp palm (motif F-E) and thumb subdomains. Apocryptovirus is placed with high confidence (100% bootstrap support) in a clade of Apocrypto-like narnaviruses, which include the bi-segmented Aspergillus lentulus narnavirus 1 (BCH36643.1), which itself are a sister-clade to the Matroyshka and Matryoshka-like narnaviruses. Bacteriophage (*Fiersviridae*) is included as an out-group. Scale bar represents 1 amino acid substitution per site.

**Supplementary Figure S5: Novel RdRp Motif Sequence Analysis.**

**A.** Conservation map of selected RdRps from “Ao-proximal” RdRp MSA. Sequences as follows (from top to bottom): Broddbo narna-like virus [UYE93831.1], Aspergillus lentulus Narnavirus 1 [BCH36645.1], Matryoshka RNA virus 3 [DAZ89878.1], Ao [SRR446909], Matryoshka RNA virus 4 [DAZ89879.1], Ao-like sp. [SRR14557243], Cane toad associated narna-like virus, [QXT57879.1], Beihai narna-like virus 21 [YP 009333140.1]. Novel and core motifs are highlighted, residues colored by chemical properties and degree of conservation. **B.** Sequence logos for novel and core motifs. **C.** Scatterplot of mean pairwise percent conservation between novel and core motifs. Pairwise percent conservation was calculated within each motif for every sequence selected. The mean conservation was calculated across the core motifs (F-E) and the novel motifs (α-μ) and plotted for each pairwise comparison. Inter-motif regions with high occupancy are also plotted in the same manner, serving as a background conservation rate. **D.** Violin plots for the difference in mean conservation for groups specified. Strongly positive/negative values indicate a large difference in conservation. Values close to zero suggest both sets of motifs are equivalently conserved.

**Supplementary Figure S6: Extended Structural Analysis.**

**A.** Predicted structure of Ao RUB RdRp. Investigating the ColabFold predictions, we note the positioning of motifs γ and η are in close proximity with the catalytic core of RdRp, with residues Arg9 of motif γ, together with Lys5 and Arg7 of motif η, are highly conserved across the Apocrypto viruses. Structurally, the three are oriented towards the catalytic site above motif C. The structure and evolutionary conservation suggests these might act as a stabilizing mechanism for the negatively-charged template RNA backbone. **B.** Secondary structure topology map for Ao core RdRp and extension domains with canonical and novel motifs highlighted. **C.** Plots for the predicted local difference distance test (pLDDT), a per-residue estimate of accuracy, for the five predicted structures and predicted alignment error for the rank one model. **D.** Distance maps for pairwise alignments of select RdRp structures against Ao. The structure for A. Neoptolemus is shown, with each residue coloured by its distance to the corresponding residue in the structural alignment against Ao RdRp. Residues that did not align are shown in white. The N’- and C’-extensions appear to be topologically unique domains, distinct from the core RdRp. The motifs identified in these regions are structurally conserved within the Apocrypto-proximal RdRps, with the N’-motifs (α-γ) more strongly conserved than the C’-ones (λ and μ). Moreover, some of these (e.g., motif β) may be conserved across all of Narnaviridae and Mitoviridae, but it is difficult to say for certain as structural alignments may be capturing similarity, and not homology at these evolutionary distances.

**Supplementary Figure S7. DGE of human macrophages infected with various T. gondii strains.**

**A.** MA plot of T. gondii - RUB vs mock genes (highlighted: Benjamini-Hochberg adjusted p-value < 0.05). **B.** Bar plot of normalized transcription counts of IFNB1 and IFNA1 across T. gondii strains and mock sequenced in Ngˆo. et al experiments, separated by batch. **C.** Heat map of Normalized Enrichment Scores (NES) from Gene Set Enrichment Analysis (GSEA) using gene sets possessing interferon-specific genes, namely IFNA1 and IFNB1, applied to the T. gondii strains. **D.** Heat map of NES values from GSEA using the Hallmark gene sets. E. GSEA curves comparing RUB vs. Mock strain using inflammatory response, cellular response to virus, and interferon-mediated signalling pathway gene sets. **F.** Volcano plot of differentially regulated genes with genes of notable gene sets being labeled.

**Supplementary Figure S8: Inspection of Differential Gene Expression data displaying batch effects.**

**A.** PCA plot of batch effects visible in Ngô et al. datasets for Human Monocytes and Neuronal Stem Cells. **B.** GSEA curves comparing Mock strains between Batches using inflammatory response, interferon alpha response, and interferon gamma response gene sets. **C.** Volcano plot of differentially regulated genes with genes of interferon alpha response, and interferon gamma response gene sets being labeled. **D.** Heat map of NES values from GSEA using the Hallmark gene sets comparing mock batch 1 vs. mock batch 2.

# Supplementary Table Legends

**Supplementary Table S1: Ngô sequencing datasets and metadata.**

Metadata describing BioProject PRJNA241125 (Ngô study) including flowcell header metadata extracted from raw (fastq) sequence headers and normalized expression (RPKM) of *Apocryptovirus odysseus* per library.

**Supplementary Table S2: *Toxoplasma gondii* strains.**

Strains of *Toxoplasma gondii* analyzed from BioProjects PRJNA241125 (Ngô study) and PRJNA114693 (Melo study), and their associated metadata.

**Supplementary Table S3**: **Apocryptoviruses and associated SRA metadata.**

**A.** Complete list of exemplar Apocryptoviruses identified in this study. **B.** Description of each Sequence Read Archive (SRA) library from which a relevant species-like operational taxonomic unit (sOTU) was identified. **C.** The pairwise percentage identity between exemplar Apocryptoviruses.
