# Supplementary figures and images for: A parasite odyssey: An RNA virus concealed in Toxoplasma gondii
Source: Virus Evol. 2024 May 11;10(1):veae040. doi: 10.1093/ve/veae040 (PMC11137675; doi:10.1093/ve/veae040)

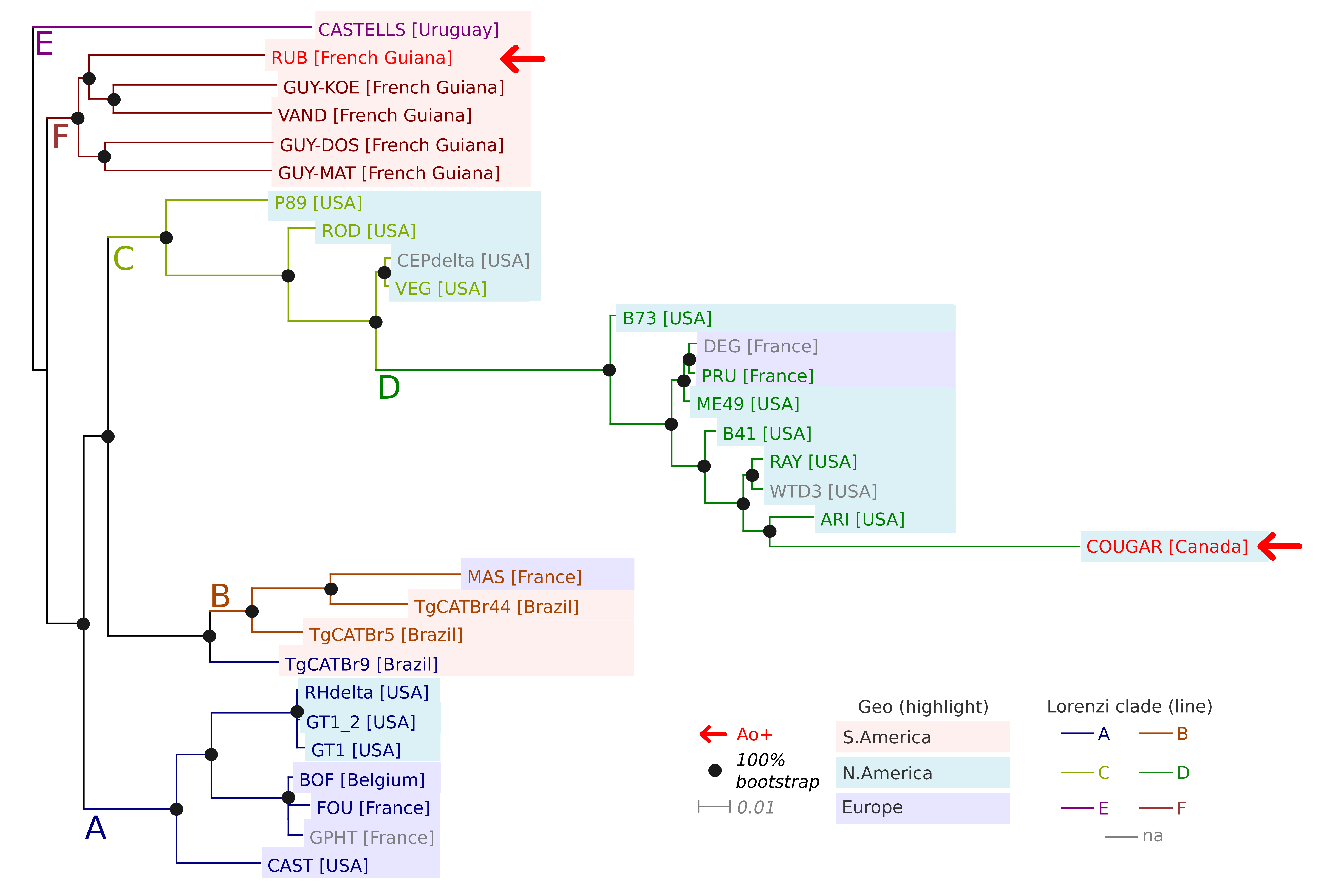

Supplement: veae040_Supp [file veae040_supp.zip › suppl_data/SF1_tgphylo.png]

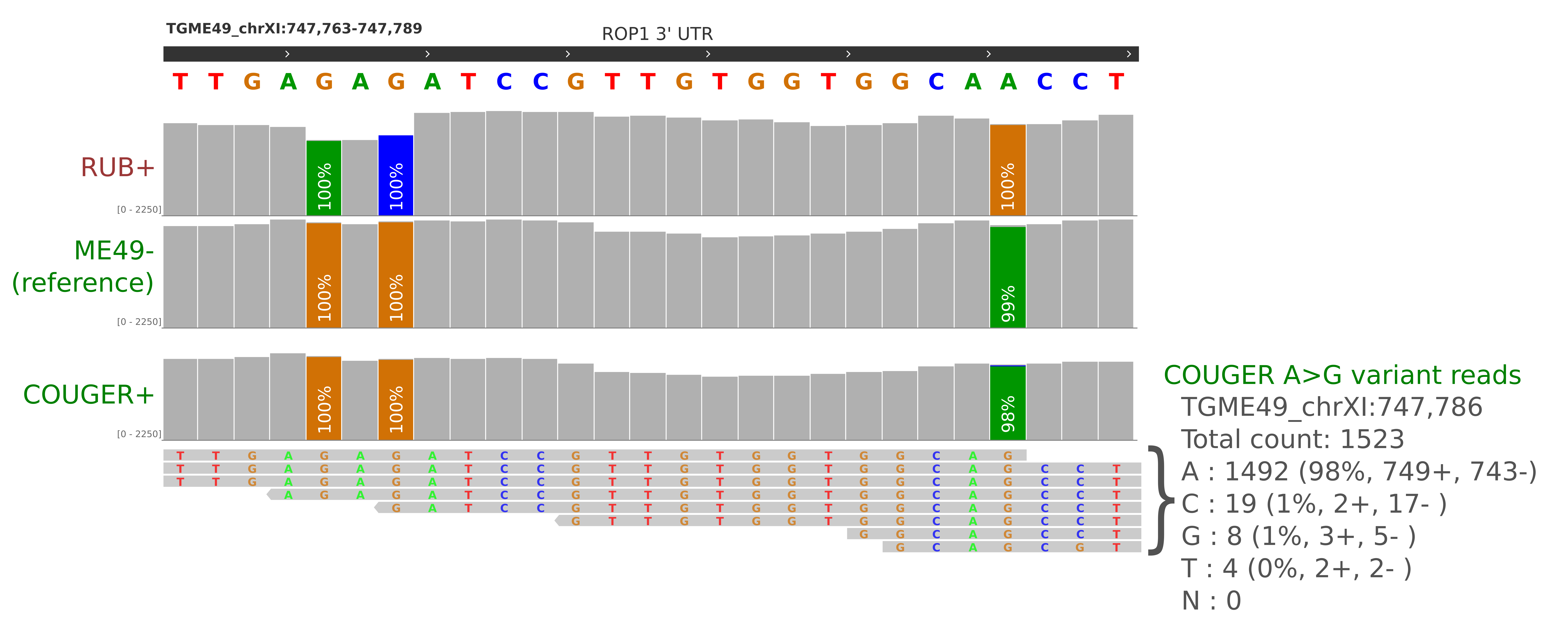

Supplement: veae040_Supp [file veae040_supp.zip › suppl_data/SF2_tg_rna_vaf.png]

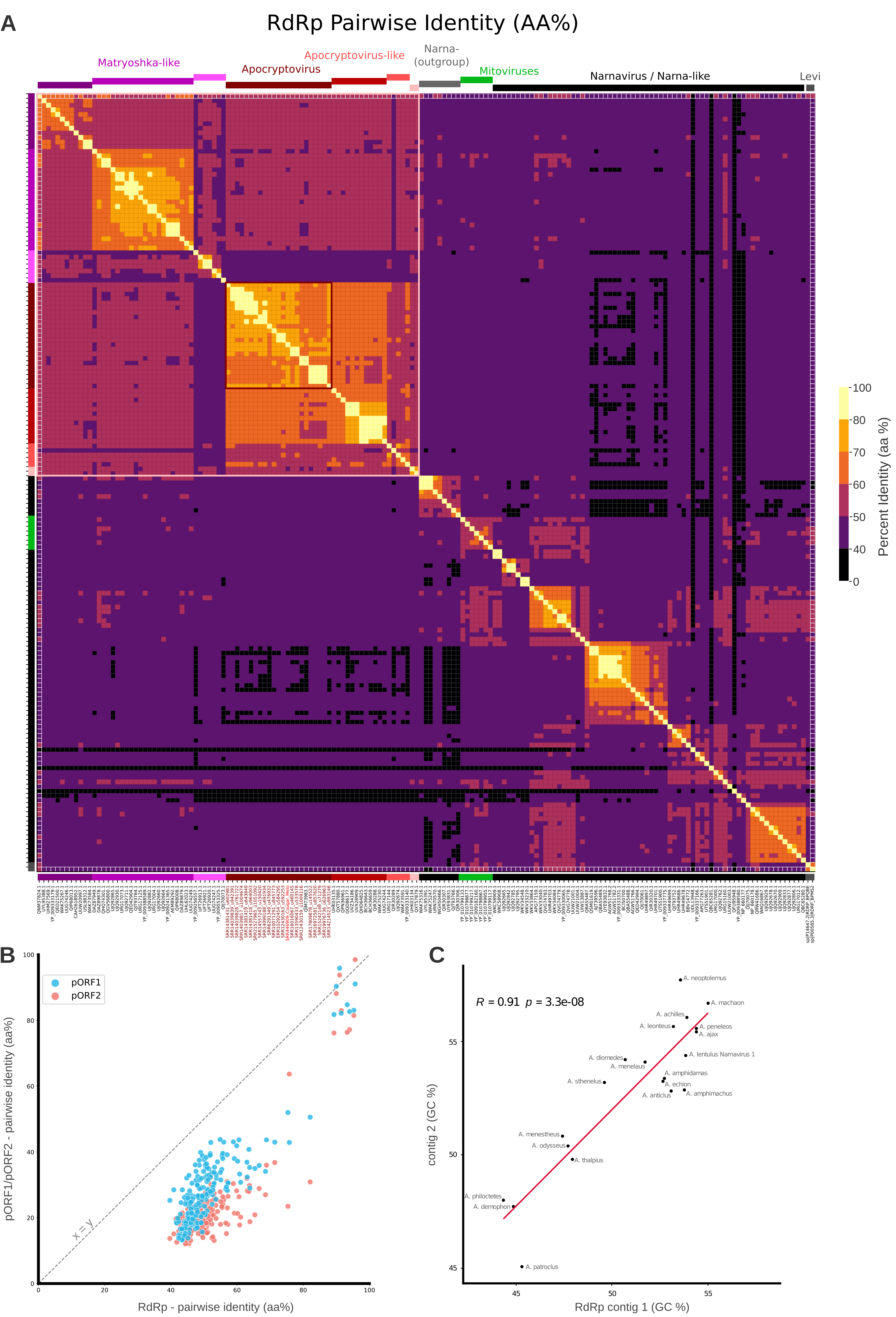

Supplement: veae040_Supp [file veae040_supp.zip › suppl_data/SF3_rdrp_pid.png]

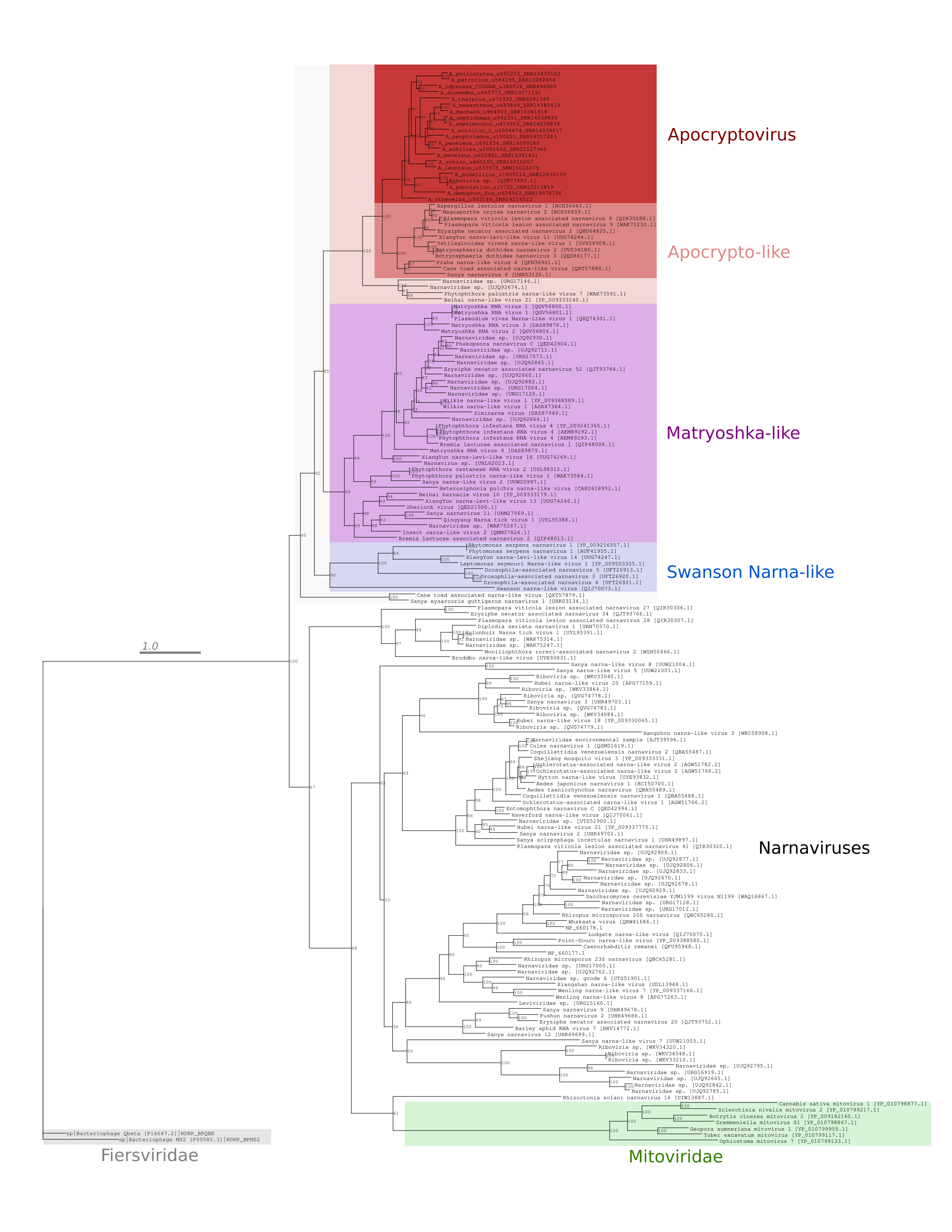

Supplement: veae040_Supp [file veae040_supp.zip › suppl_data/SF4_narnaphylo.png]

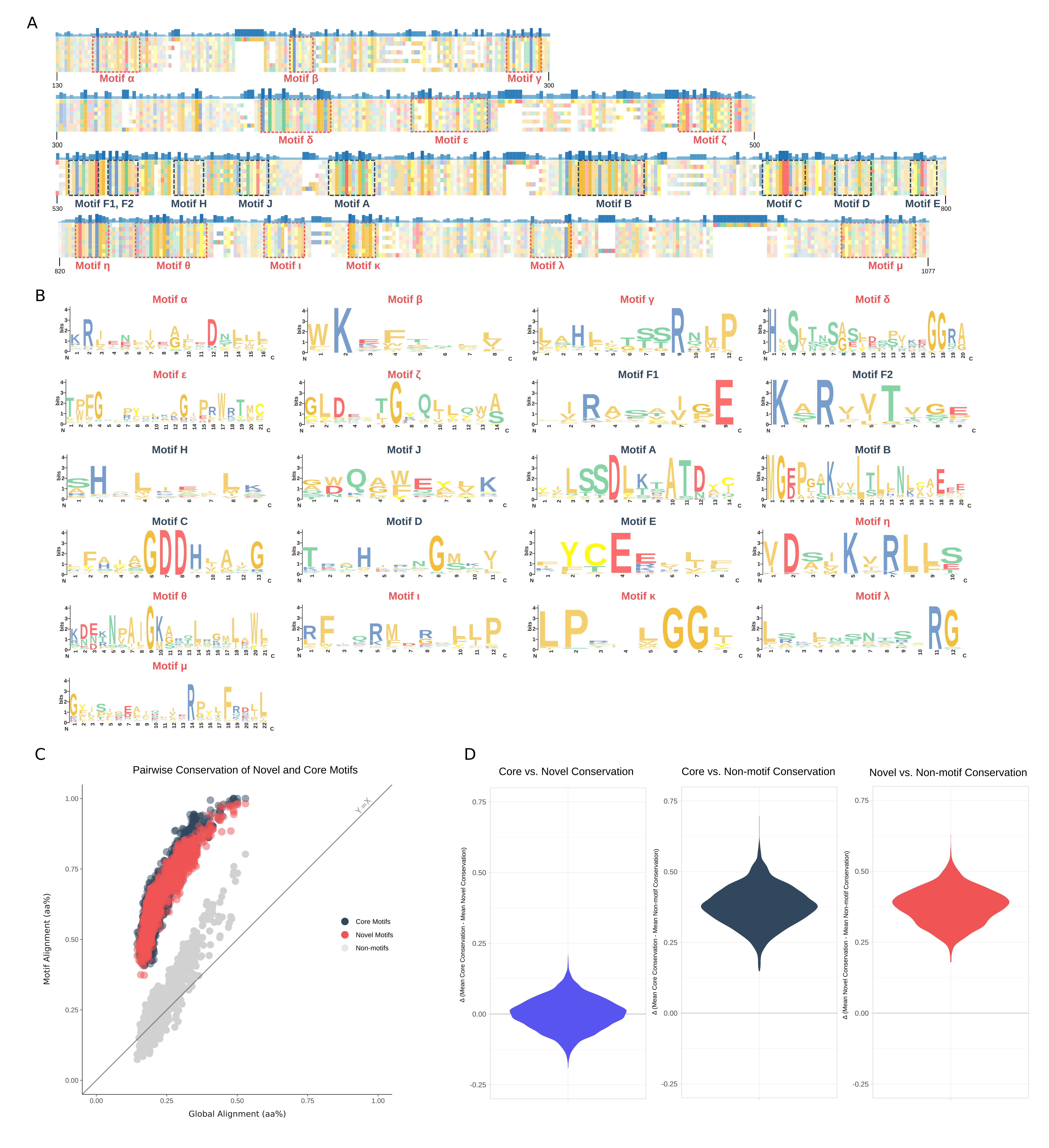

Supplement: veae040_Supp [file veae040_supp.zip › suppl_data/SF5_rdrp_motifs.png]

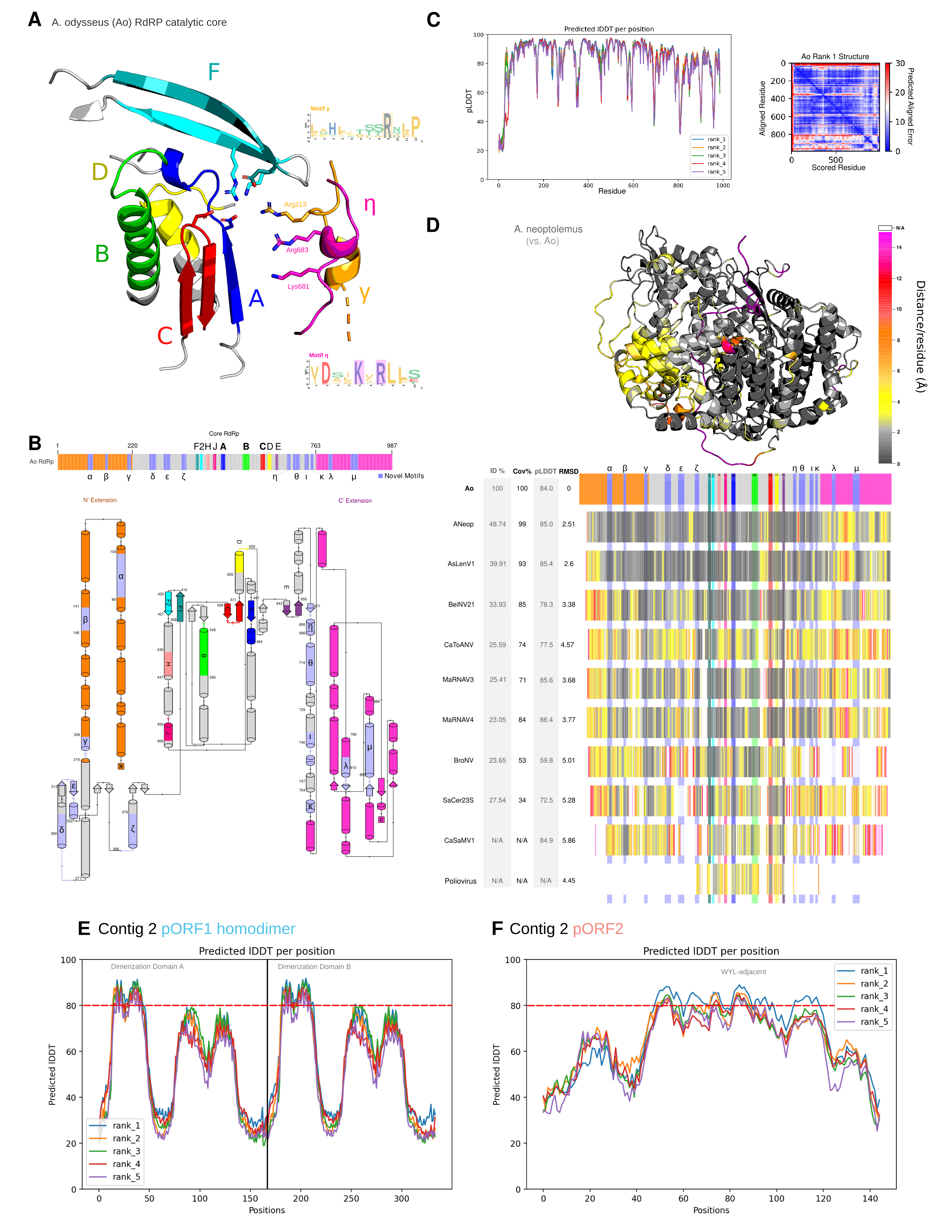

Supplement: veae040_Supp [file veae040_supp.zip › suppl_data/SF6_rdrp_struc.png]

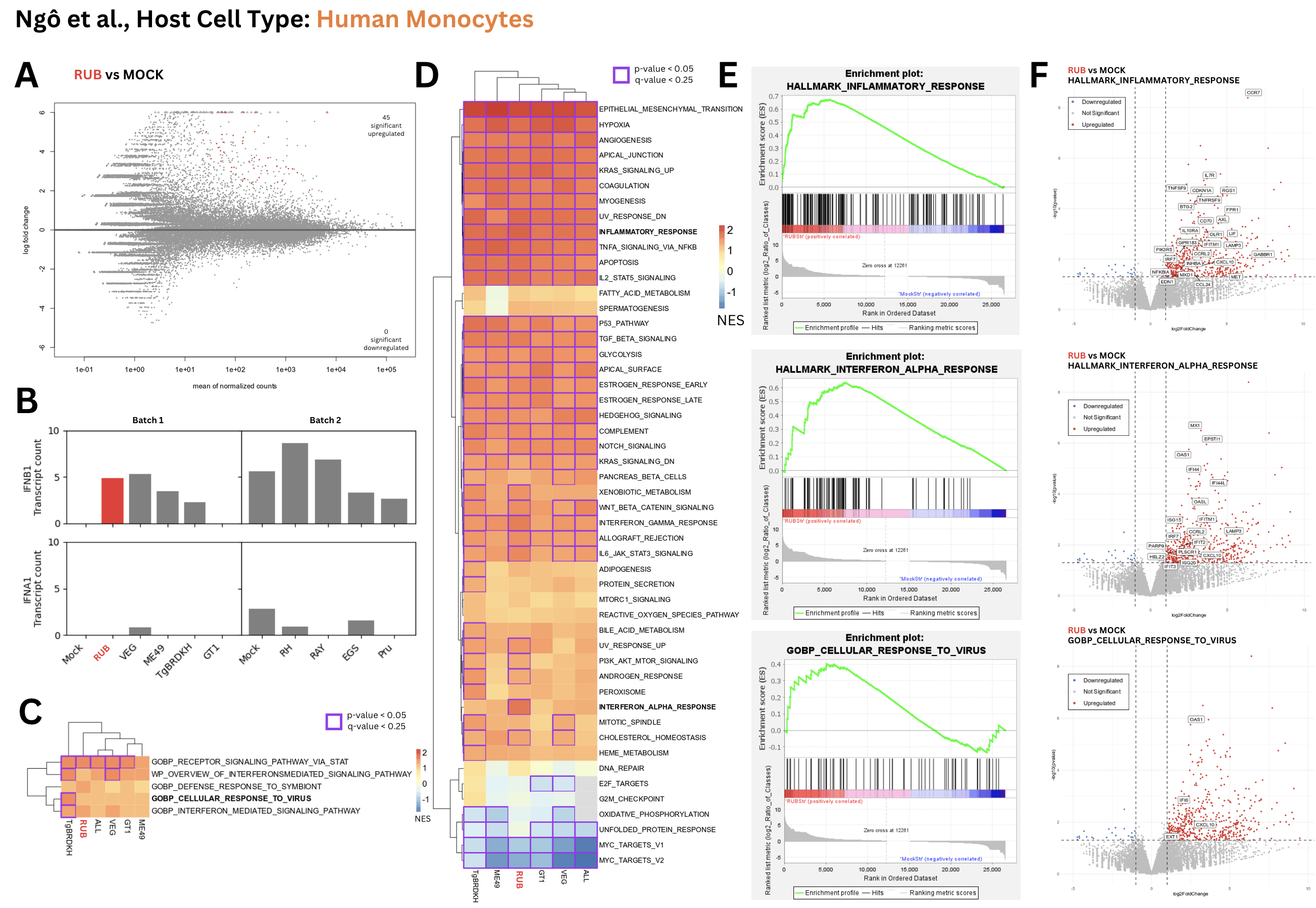

Supplement: veae040_Supp [file veae040_supp.zip › suppl_data/SF7_dge_monocyte.png]

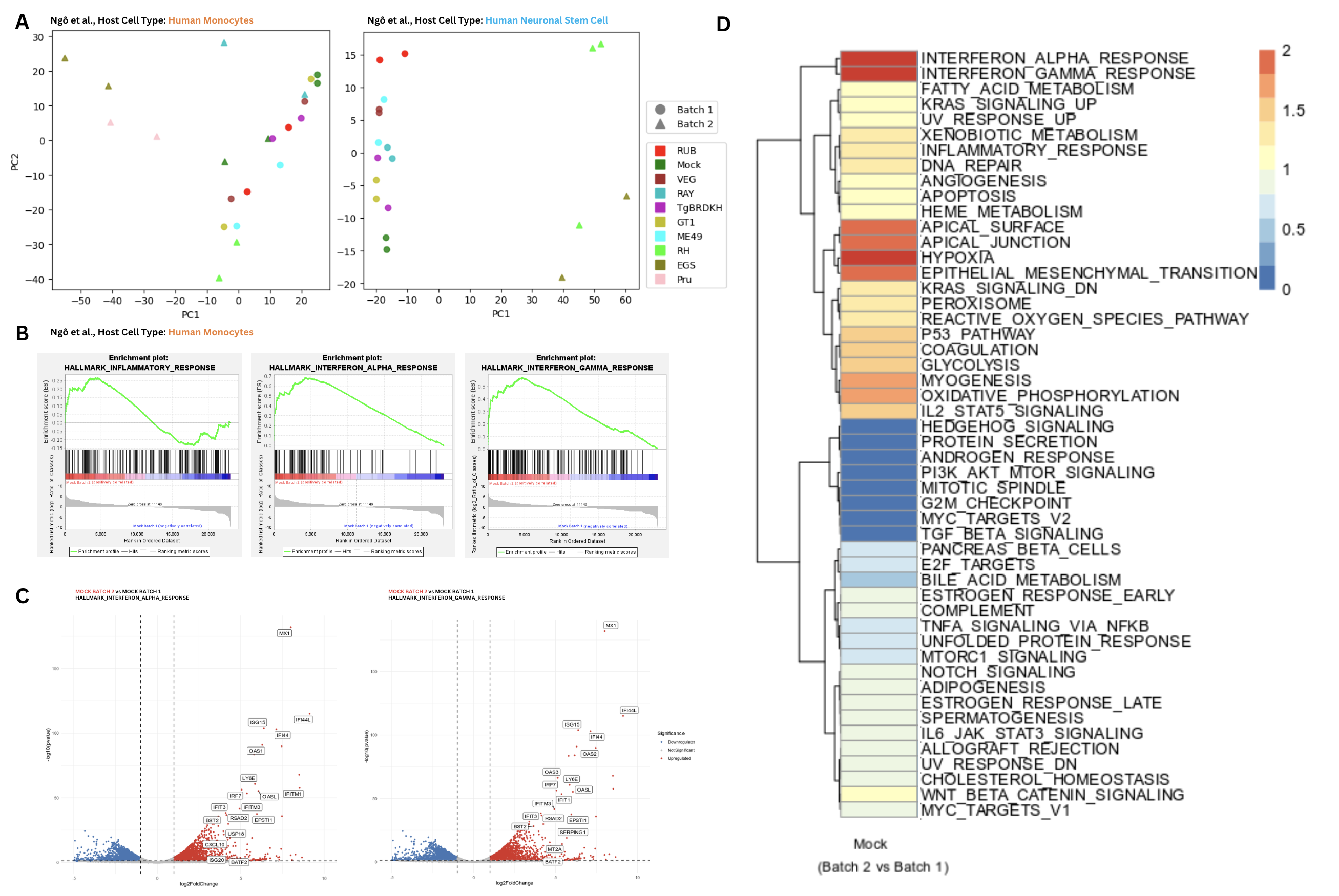

Supplement: veae040_Supp [file veae040_supp.zip › suppl_data/SF8_dge_batch.png]
